# Fibre-Reinforced Concrete at Elevated Temperatures: Damage Mechanisms, Spalling, and Residual Performance

**DOI:** 10.3390/ma19204396

**Published:** 2026-10-19

**Authors:** Jinbo Xie, Wenlong Zhao, Xi Du, Lixin Chang, Yungui Pan, Qinglong You

**Affiliations:** 1 Department of Civil Engineering, School of Environment and Architecture, University of Shanghai for Science and Technology, No. 516 Jun Gong Road, Shanghai 200093, China; 2 Shanghai Construction Management Vocational College, Shanghai 201702, China

**Keywords:** fibre-reinforced concrete, elevated temperature, steel fibre, polypropylene fibre, glass fibre, hybrid fibres, residual performance, spalling

## Abstract

Fibre-reinforced concrete exhibits complex deterioration when exposed to elevated temperatures because of physicochemical degradation, moisture migration, thermal incompatibility, pore-pressure development, and progressive cracking. Previous reviews have largely addressed temperature-dependent mechanical properties, residual performance, or spalling behaviour separately, while the connections between fibre-specific thermal mechanisms, residual mechanical performance, cracking and spalling, and hybrid-fibre interactions remain insufficiently integrated. This review addresses this gap through a mechanism-based comparison of concrete reinforced with steel, polypropylene (PP), glass, and hybrid fibres. Relevant peer-reviewed studies were identified through targeted keyword searches covering fibre-reinforced concrete, elevated-temperature exposure, residual mechanical properties, cracking, spalling, and hybrid-fibre effects, and were subsequently synthesised according to fibre type and dominant high-temperature mechanism. Based on this synthesis, distinct fibre-specific functions under elevated-temperature exposure were identified. Steel fibres primarily improve crack bridging, stress transfer, and post-cracking toughness, although their effectiveness decreases as the fibre–matrix interface deteriorates at severe temperatures. PP fibres mainly enhance vapour transport by promoting pore connectivity after thermal softening and melting, thereby reducing pore-pressure accumulation and explosive spalling. Glass fibres can restrain microcrack development and contribute to residual strength within suitable temperature ranges, but their high-temperature performance is more dependent on fibre and matrix characteristics. Hybrid fibre systems, particularly steel–PP combinations, provide complementary benefits by integrating pore-pressure relief with crack control and residual load transfer. The effects of fibre dosage and geometry, matrix properties, heating regime, moisture condition, and restraint are also discussed. The review highlights the need for fibre design strategies that balance spalling resistance, residual strength, stiffness, and post-cracking performance rather than optimizing a single property.

## 1. Introduction

Concrete is widely used in buildings and infrastructure because of its mechanical performance, durability, and inherent resistance to fire. Nevertheless, prolonged or rapid exposure to elevated temperatures can substantially alter its internal structure and mechanical behaviour. Heating induces evaporation and redistribution of internal moisture, dehydration, and decomposition of cement hydration products, differential thermal deformation between aggregates and cement paste, and progressive deterioration of the interfacial transition zone. These processes promote pore coarsening, microcracking, and loss of stiffness and strength. Under severe heating conditions, the combined effects of restricted vapour transport, pore-pressure development, and thermal stresses may further lead to surface scaling or explosive spalling, which can rapidly reduce the effective cross-section and expose the interior of structural members to fire [1,2,3]. This problem is particularly relevant to high-strength concrete (HSC), high-performance concrete (HPC) and ultra-high-performance concrete (UHPC), whose dense pore structures may restrict vapour transport despite their superior mechanical properties under ambient conditions [3,4].

The incorporation of fibres provides an effective approach for modifying the high-temperature response of concrete. However, the role of fibres cannot be described simply as an increase in strength because different fibre types act at different stages of thermal damage. Steel fibres possess relatively high stiffness and thermal stability and can continue to bridge cracks after matrix cracking, thereby improving stress transfer, limiting crack opening and preserving post-cracking capacity. In contrast, polypropylene (PP) fibres undergo thermal expansion, softening, and melting at comparatively low temperatures. The resulting interfacial damage, voids, and connected transport paths can substantially increase permeability, facilitate vapour release, and reduce the susceptibility of dense concrete to explosive spalling [5,6]. Glass fibres can also bridge thermally induced microcracks and contribute to residual integrity within suitable temperature ranges, although their effectiveness is more sensitive to fibre and interface degradation at severe temperatures, and the available evidence remains less extensive than that for steel and PP fibres [7].

These distinct mechanisms also explain why no single fibre type provides optimum performance for all high-temperature response parameters. Steel fibres generally offer greater advantages in post-cracking load transfer, flexural behaviour, and toughness, whereas PP fibres are particularly effective in controlling vapour-pressure-related spalling. Hybrid systems, especially steel–PP combinations, attempt to exploit these complementary functions. Experimental studies on UHPC have shown that PP-fibre channels can interact with thermally induced microcracks associated with steel fibres, increasing the connectivity of the transport network, and thereby improving permeability and resistance to explosive spalling [8]. At the same time, the remaining steel fibres continue to provide crack bridging and residual load-carrying capacity. Consequently, the performance of hybrid-fibre systems should be understood in terms of the interaction between transport regulation and mechanical reinforcement rather than as a simple additive effect of individual fibres.

For clarity, several thermal-exposure terms are distinguished throughout this review. “Elevated-temperature behaviour” is used as a general term for material responses under controlled thermal exposure, whereas “fire exposure” refers to tests conducted under prescribed fire curves or representative fire-heating conditions. “Hot-state properties” denote properties measured directly at the target temperature, while “residual performance” refers to properties measured after completion of heating and subsequent cooling. These categories are treated separately where the original studies provide sufficient information, because thermal history and cooling can materially affect the measured response.

Despite extensive experimental research, direct comparison among published results remains difficult. Reported high-temperature responses depend not only on fibre type but also on fibre dosage, length, diameter, and spatial distribution, as well as concrete strength, water-to-binder ratio, moisture condition, aggregate characteristics, heating rate, maximum temperature, holding duration, and cooling regime [9,10,11]. For example, PP-fibre geometry and spatial connectivity have been shown to substantially influence the permeability and explosive-spalling resistance of UHPC, while changes in heating regime can alter the apparent effectiveness of steel and PP fibres [9,10,11]. Moreover, previous reviews have generally emphasized either temperature-dependent mechanical properties of fibre-reinforced concrete, residual properties of conventional and high-strength concrete, or the spalling behaviour of UHPC [1,4,12]. Recent reviews have already addressed substantial aspects of the high-temperature behaviour of fibre-reinforced concrete. Gong et al. [13] reviewed spalling behaviour, underlying mechanisms, residual strength, and microstructural changes in FRC under elevated-temperature conditions, whereas He et al. [14] focused specifically on thermally damaged steel–PP hybrid fibre-reinforced concrete, including deterioration mechanisms, testing factors, temperature-dependent mechanical properties, and post-fire recovery. Building on these studies, the present review places greater emphasis on cross-fibre mechanistic integration across steel, PP, glass, and principal hybrid systems, linking transport regulation and crack bridging with residual mechanical response, thermal-path effects, and cross-study comparison criteria across different concrete classes. Accordingly, the contribution of this review lies primarily in providing a common mechanism-based framework for interpreting how fibre function, thermal exposure, and matrix characteristics jointly govern residual performance and spalling behaviour.

To support this comparative synthesis, relevant peer-reviewed literature was identified through targeted searches of the Web of Science Core Collection and Scopus, covering publications from 2000 to 2025. The main search terms included combinations of “fibre-reinforced concrete”, “steel fibre”, “polypropylene fibre”, “glass fibre”, “hybrid fibre”, “elevated temperature”, “fire”, “residual mechanical properties”, “cracking”, “spalling”, “pore pressure”, and “permeability”. Studies were included when they addressed fibre-reinforced concrete or closely related cementitious materials under elevated-temperature or fire exposure and provided evidence relevant to fibre-specific mechanisms, residual mechanical performance, cracking, spalling, or transport behaviour; studies unrelated to thermal exposure or lacking relevant fibre-specific information were excluded. The present article was conducted as a critical narrative review rather than a systematic review. The studies summarised in Tables 1 and 2 were selected as representative original investigations providing direct mechanistic or quantitative evidence across the principal fibre systems and thermal exposure conditions considered in this review; these tables are intended to illustrate the evidence base rather than provide an exhaustive catalogue of all retrieved studies. The final database search was conducted in May 2026.

The scope of this review is deliberately focused on steel, polypropylene (PP), glass, and their principal hybrid combinations, which provide the main evidence base for the mechanism-oriented comparisons developed here. Other fibre families, including basalt, carbon, natural, recycled-polymer, and more complex multi-hybrid systems, are not reviewed systematically and therefore fall outside the principal scope of the present synthesis. In particular, the available high-temperature evidence for glass-fibre-reinforced concrete remains substantially more limited and matrix-specific than that for steel and PP fibres; consequently, comparisons involving glass fibres are interpreted as indicative trends rather than as evidence of equivalent maturity across fibre categories.

Accordingly, this review examines the behaviour of steel-, PP-, glass- and hybrid-fibre-reinforced concrete subjected to elevated temperatures. The discussion begins with the fundamental physicochemical, thermal and hygral damage mechanisms of concrete and then compares the distinct roles of different fibre types. Particular attention is given to residual compressive, tensile and flexural properties, elastic modulus, toughness, and fracture behaviour, together with fibre-dependent cracking and spalling resistance. The effects of thermal exposure conditions, fibre architecture, and matrix characteristics are subsequently examined to explain the variability among reported results. Finally, current limitations and future research needs are discussed, with emphasis on standardized testing, quantitative characterization of fibre-related transport and crack networks, multi-objective hybrid-fibre design, and the transfer of material-scale findings to structural fire scenarios. Rather than identifying a universally superior fibre, the review aims to clarify how different fibre mechanisms can be selected and combined to balance spalling resistance, residual load-bearing capacity, and post-cracking performance.

## 2. Damage Mechanisms of Concrete at Elevated Temperatures

**Figure 1:**
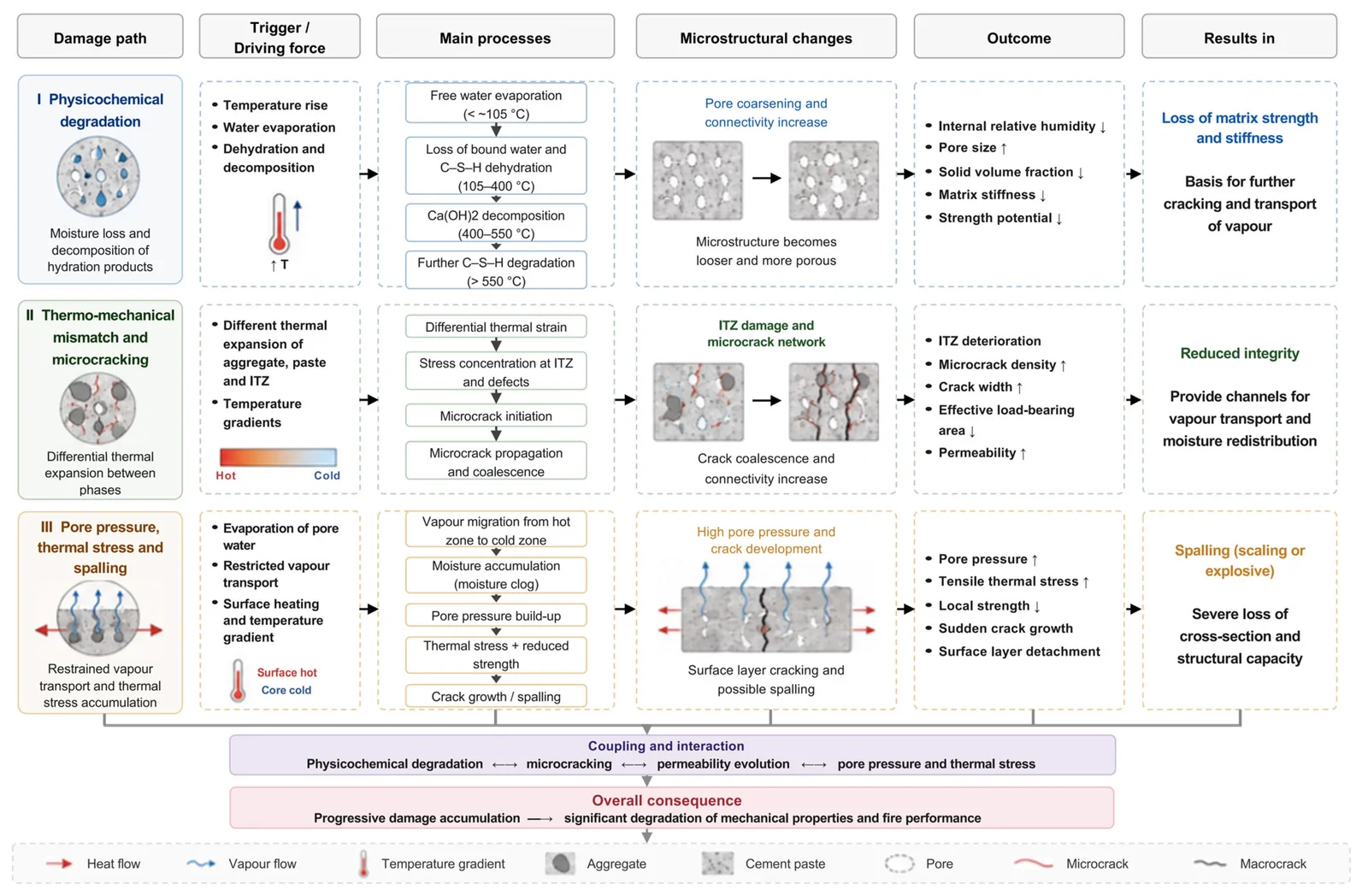
Schematic illustration of the damage mechanisms of concrete subjected to elevated temperatures.

The deterioration of concrete at elevated temperatures is not governed by a single process but arises from the combined effects of moisture transport, physicochemical transformations of the cementitious matrix, incompatible thermal deformation among different constituents, and the development of pore pressure and thermal stresses. As temperature increases, the cement paste progressively undergoes dehydration and decomposition, while the mismatch in thermal deformation among aggregates, cement paste, and the interfacial transition zone (ITZ) becomes increasingly pronounced. These processes are accompanied by pore-structure coarsening, interfacial deterioration, and the initiation and propagation of microcracks. When the transport of internally generated water vapour is restricted, pore pressure may act synergistically with thermal stresses, resulting in severe cracking and, in extreme cases, explosive spalling. Accordingly, the macroscopic degradation of concrete at elevated temperatures can be regarded as a progressive damage process governed by strongly coupled thermal, hygral, and mechanical mechanisms, as schematically illustrated in Figure 1 [15,16,17].

### 2.1. Physicochemical Changes at Elevated Temperatures

Concrete contains free water, physically adsorbed water, and chemically bound water within hydration products. Consequently, the earliest changes during heating are primarily associated with moisture evaporation and redistribution. As temperature increases, free water within capillary and larger pores progressively evaporates and migrates towards cooler regions or the external environment under temperature and vapour-pressure gradients. With further heating, adsorbed water and part of the chemically bound water are gradually released, inducing shrinkage of the cement paste and altering its original pore structure [15,16].

In addition to moisture loss, the principal hydration products within the cementitious matrix undergo varying degrees of dehydration and decomposition as temperature rises. Calcium silicate hydrate (C–S–H) progressively loses water over a broad temperature range, accompanied by changes in its nanostructure, stiffness, and bonding state. In contrast, Ca(OH)_2_ undergoes pronounced dehydroxylation mainly within the temperature range of approximately 400–550 °C. At still higher temperatures, the C–S–H structure deteriorates further, leading to enlarged pore sizes, increased pore connectivity, and more extensive microcracking within the hardened cement paste [15,16]. Through microstructural characterisation of hardened cement paste, Peng and Huang [15] demonstrated that elevated temperatures markedly modify the pore structure and accelerate the decomposition of hydration products. DeJong and Ulm [16] further revealed the temperature-dependent evolution of the structure and mechanical properties of C–S–H at the nanoscale.

Aggregates are likewise not completely stable at elevated temperatures. Different mineralogical constituents exhibit distinct coefficients of thermal expansion and phase-transition characteristics. For example, quartz-bearing aggregates may undergo the α- to *β*-quartz transformation at approximately 573 °C, resulting in a pronounced volumetric change, whereas some carbonate aggregates may thermally decompose at higher temperatures. Such transformations further intensify the mismatch in thermal deformation between the aggregate and the surrounding cement paste, thereby promoting interfacial damage. The physicochemical deterioration of concrete at elevated temperatures therefore cannot be attributed solely to degradation of the cement paste; rather, it results from the concurrent evolution of the paste, aggregates, and ITZ [17].

These physicochemical changes ultimately drive the internal structure of concrete from a relatively dense state towards an increasingly porous and cracked configuration. Moisture loss and decomposition of hydration products alter both the solid-phase volume and intrinsic mechanical properties, while pore coarsening and microcrack development further reduce the effective load-bearing area. As these effects accumulate, the elastic modulus, strength, and overall integrity of concrete progressively deteriorate, thereby facilitating subsequent thermally induced cracking and macroscopic failure [15,16,17].

### 2.2. Microcracking and Interfacial Damage

Concrete is a typical heterogeneous multiphase composite consisting primarily of cement paste, fine and coarse aggregates, and interfacial transition zones. These constituents differ in their coefficients of thermal expansion, elastic moduli, and temperature sensitivity and therefore undergo incompatible deformation during heating. Even when the macroscopic temperature field is relatively uniform, thermal-expansion mismatch between aggregates and the surrounding cement paste can generate substantial local stress concentrations, with the ITZ and pre-existing defects generally representing the most vulnerable regions [18].

Through mesoscopic thermoelastic analysis, Fu et al. [18] demonstrated that the mismatch in thermal expansion between aggregates and the cementitious matrix can induce substantial local tensile stresses near the interfaces, thereby promoting microcrack initiation. Under actual heating conditions, this thermomechanical effect interacts with dehydration-induced shrinkage of the cement paste, temperature-dependent stiffness degradation, and local thermal gradients, causing cracks to progressively propagate from interfacial regions into the surrounding cementitious matrix.

At relatively low levels of thermal damage, cracks generally remain dispersed as isolated microcracks, and the material retains a comparatively high degree of structural continuity. With increasing temperature, however, both the number and width of microcracks increase, and neighbouring cracks progressively interact, coalesce, and eventually form interconnected crack networks. Crack propagation reduces the effective load-bearing area and overall stiffness of concrete while simultaneously impairing stress transfer across the aggregate–paste interfaces. Interfacial deterioration and crack coalescence therefore constitute important mesoscopic origins of the loss of strength and stiffness after exposure to elevated temperatures [17,18].

Crack development, in turn, alters moisture and gas transport during heating. On the one hand, increasing crack density creates additional transport pathways and enhances the permeability of the material. On the other hand, before a sufficiently interconnected crack network is established, local moisture may continue to migrate and accumulate under thermal gradients. Cracking is therefore not only a consequence of thermal damage but also an important factor governing the subsequent evolution of internal pore pressure.

From this perspective, the evolution of microstructural damage at elevated temperatures should be regarded as a coupled and self-reinforcing process. Physicochemical degradation and differential thermal deformation promote interfacial microcracking, while subsequent crack propagation and coalescence progressively modify the permeability and transport properties of concrete. These changes, in turn, affect moisture redistribution and pore-pressure development during continued heating, thereby further influencing the evolution of thermal and hygral damage. Therefore, high-temperature cracking cannot be attributed solely to thermal-expansion mismatch or decomposition of the cementitious matrix. Rather, it results from the continuous interaction among physicochemical degradation, thermally induced deformation incompatibility, crack evolution, and internal moisture and gas transport.

### 2.3. Pore Pressure, Thermal Stress, and Spalling

High-temperature spalling is among the most destructive failure phenomena experienced by concrete under fire exposure and has attracted particular attention in studies of high-strength concrete (HSC) and high-performance concrete (HPC). During heating, internal moisture evaporates and generates water vapour. Owing to pronounced temperature and pressure gradients within the material, vapour migrates from hotter regions towards cooler regions and eventually towards the external environment. When permeability is low or internal transport pathways are insufficient, vapour migration becomes restricted, potentially resulting in substantial local pore-pressure build-up [2,19].

Through simultaneous measurements of temperature and pore pressure in HPC, Kalifa et al. [2] identified pronounced pore-pressure peaks during heating and demonstrated that their location was closely associated with the thermal field and moisture transport. These observations established pore pressure as an important contributor to thermally induced spalling. Subsequent studies, however, have shown that spalling cannot be adequately explained by a pore-pressure mechanism alone.

As water vapour migrates from hotter regions towards relatively cooler regions, part of the transported moisture may condense and form a locally water-saturated or highly moist zone. This phenomenon is commonly referred to as moisture clogging. Chen et al. [20], through gas-permeability measurements of cementitious materials under elevated-temperature conditions, provided experimental evidence for this moisture-clogging effect. Once a local high-moisture region develops, further gas transport may be hindered, allowing vapour pressure to accumulate near the drying front. By simultaneously monitoring temperature, mass loss, and pore pressure, Mindeguia et al. [19] further demonstrated the dynamic interdependence among moisture transport, material dehydration, and pore-pressure development.

Internal pore pressure, however, represents only one important component of the spalling process. Under realistic fire exposure or one-sided heating, the concrete surface generally heats much faster than the interior, resulting in steep thermal gradients. The heated surface layer tends to expand, while its deformation is constrained by the relatively cooler interior, thereby producing a complex thermally induced stress state. At the same time, the tensile strength, fracture energy, and stiffness of concrete progressively decrease with increasing temperature. When thermal stresses and pore pressure act in combination with the continuously deteriorating fracture resistance of the material, local cracks may rapidly propagate and cause detachment of the surface layer; under severe conditions, this process manifests as explosive spalling [2,21].

Felicetti et al. [21] investigated the influence of pore pressure on the fracture behaviour of heated concrete using specially designed high-temperature fracture tests. Their results indicated that pore pressure can substantially promote fracture under thermal exposure, while also demonstrating that a simple criterion based solely on pore pressure exceeding the tensile strength of concrete is insufficient to fully describe the spalling process. A more physically meaningful interpretation is therefore to regard high-temperature spalling as a coupled thermo-hygro-mechanical failure process, rather than as a failure mode controlled by a single physical mechanism.

Spalling susceptibility is additionally influenced by numerous material and testing parameters, including the initial moisture content, matrix permeability, concrete strength class, specimen dimensions, heating rate, external restraint, and applied stress state. This issue is particularly important for HSC and HPC. Their low water-to-binder ratios and dense pore structures are advantageous for mechanical strength and durability at ambient temperature, but simultaneously restrict the rapid escape of water vapour during heating and can therefore promote pore-pressure accumulation under certain conditions [2,19,22].

Using neutron imaging, Toropovs et al. [22] monitored the evolution of temperature, pressure, and moisture distribution in HPC during heating in real time, providing further insight into the dynamic redistribution of moisture and the associated development of pore pressure. Such observations demonstrate that the various high-temperature damage mechanisms do not occur independently in a simple sequential manner. Instead, moisture transport, thermal fields, cracking, and permeability continuously interact through mutually reinforcing feedback mechanisms.

Overall, as illustrated in Figure 1, the damage mechanisms of concrete at elevated temperatures can be classified into three major, strongly coupled pathways. First, moisture evaporation together with dehydration and decomposition of hydration products causes physicochemical degradation of the cementitious matrix and promotes pore-structure coarsening. Second, incompatible thermal deformation among aggregates, cement paste, and the ITZ drives the initiation, propagation, and coalescence of microcracks. Third, pore-pressure build-up caused by restricted moisture transport interacts with thermally induced stresses associated with temperature gradients and, in conjunction with the progressive loss of fracture resistance, may result in severe cracking and spalling. These mechanisms interact continuously and ultimately govern the macroscopic mechanical degradation and failure modes of concrete subjected to elevated temperatures.

Different fibre types can intervene at distinct stages of this damage sequence. Steel fibres primarily contribute to crack bridging and post-cracking stress transfer, whereas polypropylene (PP) fibres primarily modify moisture and vapour transport conditions at elevated temperatures. Glass fibres, in turn, can contribute to microcrack control within an appropriate temperature range. A clear understanding of the intrinsic high-temperature damage mechanisms of plain concrete therefore provides the essential basis for analysing the mechanisms by which different fibres modify its thermal response and for explaining their contrasting effectiveness under elevated-temperature exposure.

## 3. Behaviour and Mechanisms of Different Fibres at Elevated Temperatures

The incorporation of fibres can substantially modify crack propagation, moisture and vapour transport, pore-pressure development, and post-cracking stress transfer in concrete exposed to elevated temperatures. However, different fibre types differ markedly in material composition, elastic modulus, thermal-expansion behaviour, melting or softening characteristics, and fibre–matrix interfacial stability. Consequently, their mechanisms of action under thermal exposure are fundamentally different. In general, steel fibres primarily preserve the integrity of heated concrete through crack bridging and stress transfer; polypropylene (PP) fibres mainly modify pore connectivity and vapour-transport conditions through thermal expansion, softening, melting, and interfacial damage; and glass fibres contribute mainly to microcrack bridging and crack control, thereby maintaining a certain degree of residual integrity. Hybrid-fibre systems further exploit the complementary mechanisms of different fibre types to simultaneously regulate pore-pressure development, crack propagation, and residual load-bearing capacity [7,8,23,24].

**Figure 2:**
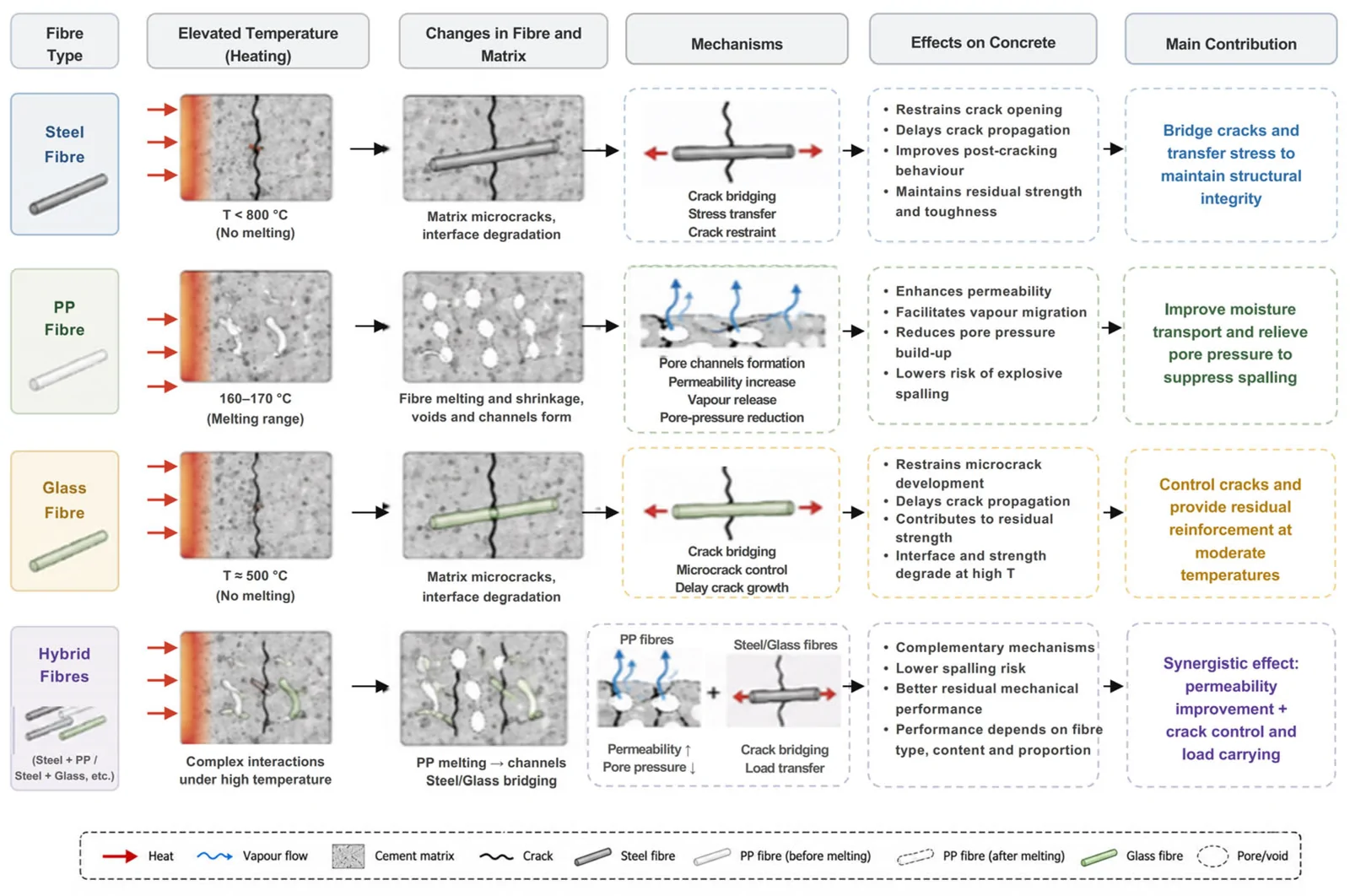
Dominant mechanisms of steel, polypropylene, glass, and hybrid fibres in concrete subjected to elevated temperatures.

Accordingly, the high-temperature performance of different fibres should not be reduced to a simple comparison of which fibre type performs “best”. Steel, PP, and glass fibres act at different stages of the thermal-damage process, while their relative effectiveness is further influenced by concrete type, fibre content and geometry, heating regime, and initial moisture condition. The dominant mechanisms associated with steel, PP, glass, and hybrid fibres under elevated-temperature exposure are schematically summarised in Figure 2. Understanding these mechanistic differences is therefore essential for interpreting the substantial variations in residual strength, toughness, and spalling behaviour reported in the literature.

### 3.1. Steel Fibres

Steel fibres are characterised by high elastic modulus, tensile strength, and thermal stability. Under conventional fire exposure and most laboratory heating conditions, steel fibres do not undergo melting in the same manner as polymeric fibres. Consequently, when cracking develops in the cementitious matrix as a result of dehydration, thermal-expansion mismatch, and interfacial deterioration, steel fibres crossing the cracks can continue to transfer tensile stresses and restrain crack opening and further propagation through bridging action. Mechanically, this effect redistributes localised stresses near crack tips to the surrounding matrix and adjacent fibres, thereby delaying the rapid transition from isolated cracking to macrocrack formation [1]. Studies on steel-fibre-reinforced reactive powder concrete (RPC) and other high-strength cementitious composites have consistently shown that steel fibres are particularly effective in modifying post-cracking failure behaviour and energy-dissipation mechanisms under elevated temperatures.

However, it is inaccurate to describe the role of steel fibres simply on the basis that they “do not melt and therefore always enhance concrete at elevated temperatures”. Their ability to bridge cracks effectively depends strongly on the bond condition between the fibre and the surrounding matrix. Load transfer in steel-fibre-reinforced concrete is generally governed by a combination of chemical adhesion, friction, and mechanical anchorage provided by hooked ends, corrugations, or surface deformation. As temperature increases, dehydration of the cement matrix, pore coarsening, and interfacial cracking progressively weaken these bonding mechanisms, eventually promoting fibre slip, interfacial debonding, or pull-out [25,26].

Abdallah et al. [25] investigated the elevated-temperature pull-out behaviour of straight and hooked-end steel fibres embedded in cementitious matrices of different strength levels and demonstrated that both fibre geometry and matrix strength strongly affect the post-heating interfacial bond response. Ruano et al. [26] further linked single-fibre pull-out behaviour to the residual macroscopic performance of steel-fibre-reinforced high-strength concrete, showing that the deterioration of toughness and post-cracking performance after thermal exposure cannot be attributed solely to damage of the cementitious matrix but is also directly associated with degradation of the fibre pull-out mechanism.

From a mesoscopic perspective, the high-temperature reinforcing efficiency of steel fibres therefore depends on the combined retention of fibre mechanical stability, fibre–matrix bond, and mechanical anchorage. All three components may deteriorate to different extents with increasing temperature. This is particularly relevant for hooked-end, corrugated, and multi-anchored steel fibres. Although stronger mechanical anchorage generally improves post-cracking energy dissipation at ambient temperature, high-temperature matrix softening and interfacial degradation can substantially modify the governing pull-out mechanism. Recent studies on 3D, 4D, and 5D steel fibres further demonstrate that fibre geometry has a pronounced influence on residual strength and toughness after thermal exposure, indicating that different steel-fibre configurations should not be treated as having equivalent high-temperature reinforcing efficiency [27].

The role of steel fibres in spalling resistance should also be interpreted cautiously. Steel fibres can restrain rapid crack propagation and may alter local permeability through thermally induced microcracking around the fibres. Nevertheless, their principal function remains crack bridging and post-cracking stress transfer rather than the direct creation of extensive vapour-release pathways, as occurs with PP fibres. Therefore, in dense high-strength concrete (HSC) and ultra-high-performance concrete (UHPC), the incorporation of steel fibres alone does not necessarily eliminate the risk of explosive spalling [11,28].

### 3.2. Polypropylene Fibres

The high-temperature behaviour of PP fibres differs fundamentally from that of steel fibres. PP fibres exhibit relatively low softening and melting temperatures, with melting generally occurring at approximately 160–170 °C. During heating, PP fibres initially undergo thermal expansion and relative deformation with respect to the surrounding matrix, followed by softening, shrinkage, and eventual melting. These processes modify the interfacial region around the fibres and promote the connection of pores and microcracks within the cementitious matrix [3,5].

Early explanations of the beneficial role of PP fibres commonly emphasised that melting fibres leave behind empty channels. Although this interpretation is intuitive, it does not fully account for the experimental evidence. Kalifa et al. [3] showed that PP fibres can markedly reduce internal pore pressure in heated HPC while substantially increasing material permeability after thermal exposure. Liu et al. [5] further demonstrated that thermally induced interfacial microcracking, fibre melting, and changes in the fibre–matrix interfacial zone jointly contribute to spalling mitigation. The critical role of PP fibres therefore lies not in the formation of isolated voids, but in enhancing the effective connectivity of the pore and crack network [3,5].

During heating, thermal mismatch between PP fibres and the surrounding matrix can initiate local interfacial defects even before complete melting occurs. Subsequent softening and melting further open these regions and connect previously isolated pores and microcracks. As the transport network becomes more continuous, vapour permeability increases, internal moisture can migrate more readily, and pore-pressure accumulation is reduced. The effectiveness of PP fibres in mitigating explosive spalling should therefore be interpreted as the result of a progressive permeability-enhancement process rather than a simple consequence of fibre melting alone.

Zeiml et al. [6], through permeability and pore-structure measurements on concrete under realistic heating conditions, demonstrated that PP fibres substantially increase the permeability of heated concrete and linked this permeability enhancement to spalling behaviour. Bošnjak et al. [29] further conducted in situ permeability measurements at elevated temperatures and observed that the permeability of PP-fibre-reinforced concrete may increase sharply at temperatures considerably below the complete melting temperature of the fibres. This finding suggests that interfacial microcracking and thermal mismatch can become relevant to mechanical behaviour and transport processes before full fibre melting occurs, thereby challenging the simplified assumption that the anti-spalling effect begins only after melting.

The effectiveness of PP fibres is also governed by more than fibre mass or volume fraction. Li et al. [9] systematically investigated the effects of PP-fibre geometry and dosage on the permeability of UHPC after thermal exposure and showed that fibre length, diameter, and volume fraction jointly control the resulting microcrack network and transport capacity. Zhang et al. [10], using X-ray computed tomography, further characterised the three-dimensional spatial distribution of PP fibres in UHPC and demonstrated that aspect ratio, number density, and spatial connectivity strongly influence the formation of an effective percolating network [9,10].

Accordingly, a single fixed “recommended PP-fibre dosage” cannot be regarded as universally applicable to all concrete systems. At an identical fibre content, relatively coarse or short fibres, or fibres exhibiting pronounced agglomeration, may provide insufficient number density and spatial connectivity. Conversely, finer fibres with larger aspect ratios may create a larger number of potential interfacial transport paths even at lower volume fractions. The optimisation of PP-fibre systems should therefore consider not only dosage but also fibre geometry, number density, spatial distribution, and the resulting permeability characteristics [9,10].

The high-temperature role of PP fibres is inherently dual in nature. Increased pore connectivity and permeability are generally beneficial for spalling mitigation; however, voids left after fibre melting and thermally induced interfacial deterioration may reduce the effective load-bearing area and stiffness of the residual matrix. PP fibres should therefore not be regarded simply as high-temperature reinforcing fibres. A more accurate functional description is that they act primarily as modifiers of moisture transport and spalling behaviour. Whether they also improve residual mechanical strength depends on the exposure temperature, fibre content, and matrix characteristics.

### 3.3. Glass Fibres

In contrast to PP fibres, the primary high-temperature role of glass fibres does not rely on melting-induced pressure-release channels but is associated mainly with crack bridging and microcrack control. At low to intermediate temperatures, glass fibres can bridge microcracks within the matrix and transfer tensile stresses, thereby limiting crack opening and coalescence. From a mechanical perspective, their function is therefore closer to that of steel fibres than to that of PP fibres, although substantial differences exist in thermal stability, elastic modulus, and fibre–matrix interfacial durability.

Raza et al. [30] investigated RPC containing different glass-fibre contents after exposure to 200, 400, 600, and 800 °C. Their results showed that glass fibres could improve residual integrity and reduce severe spalling in the investigated RPC system, while the fibre content associated with improved performance varied with exposure temperature. This indicates a pronounced temperature–dosage interaction and suggests that there is no single optimum glass-fibre content applicable across the entire temperature range.

At moderate temperatures, glass fibres can contribute to the stabilisation of thermally induced microcracks by maintaining bridging stresses across developing cracks. As temperature increases further, however, changes in the fibres themselves, their surface condition, and the fibre–matrix interface progressively reduce their stress-transfer efficiency. Consequently, the reinforcing contribution of glass fibres tends to diminish under severe thermal exposure.

It is therefore inappropriate to infer their high-temperature effectiveness solely from their relatively high tensile strength at ambient temperature. Their actual contribution after heating depends on fibre type, surface treatment, fibre length and dosage, as well as the severity of thermal degradation of the surrounding matrix.

Another important limitation is that the literature on glass-fibre-reinforced concrete at elevated temperatures remains considerably less extensive than that available for steel and PP fibres. A substantial proportion of existing studies focuses on specialised matrices such as RPC and self-compacting concrete, and the resulting observations cannot necessarily be generalised directly to normal-strength concrete, HSC, or UHPC. Kumari et al. [7], for example, investigated steel–glass hybrid self-compacting concrete subjected to repeated thermal cycles and exposure temperatures of 200–600 °C and showed that the contribution of glass fibres varies substantially with thermal history.

Glass fibres are therefore more appropriately regarded as fibres capable of maintaining material integrity through crack bridging and crack refinement within a limited temperature range, with their effectiveness showing a comparatively strong dependence on the specific material system. This distinction is important when comparing glass fibres with steel fibres in subsequent discussions of residual mechanical performance.

### 3.4. Hybrid Fibres

High-temperature damage in concrete involves several interacting processes, including moisture migration, pore-pressure development, thermally induced stress, crack initiation, and post-cracking load transfer. A single fibre type is therefore unlikely to provide optimum control over all stages of the damage process. The fundamental rationale for hybrid-fibre design is not simply to increase the total fibre content, but to combine fibres with different high-temperature functions so that they act on different damage mechanisms.

Among the various hybrid systems, steel–PP fibre combinations exhibit the clearest mechanistic complementarity. PP fibres primarily modify the internal transport network by inducing interfacial damage, softening, and melting, thereby increasing pore connectivity and facilitating vapour release. Steel fibres, in contrast, remain mechanically active after crack initiation and continue to contribute to crack bridging, stress redistribution, and post-cracking load transfer. The two fibre types therefore intervene in different but complementary stages of thermal damage: PP fibres primarily regulate pressure development and transport, whereas steel fibres primarily preserve structural continuity after cracking.

Chen and Liu [23] were among the earlier researchers to investigate the residual performance of hybrid-fibre-reinforced HSC after high-temperature exposure and suggested that combining low-melting and high-melting fibres can provide a more balanced thermal response than the use of either fibre type alone. Subsequent studies by Pliya et al. [24] and Yermak et al. [11] further demonstrated the complementary roles of steel and PP fibres in spalling resistance, transport behaviour, and residual mechanical performance [11,23,24].

More importantly, the contribution of steel fibres is not necessarily restricted to mechanical bridging after PP fibres have formed transport channels. Microstructural observations by Li et al. [8] on steel–PP hybrid UHPC indicated that thermally induced microcracks around steel fibres may enhance the connectivity between voids or channels created by PP-fibre melting, thereby further increasing matrix permeability. This suggests that a genuine mechanistic interaction may develop between the two fibre types, in which PP-fibre channels and steel-fibre-induced microcracking jointly promote the formation of a more interconnected transport network.

Such an interpretation is more physically meaningful than the simplified description that “PP fibres prevent spalling while steel fibres improve strength”, because it recognises that mechanical and transport mechanisms can interact during heating.

Hybridisation, however, does not necessarily result in positive synergy. Increasing the total fibre content may reduce fresh-state workability and promote fibre agglomeration. Excessive PP-fibre contents may generate excessive porosity after melting, whereas high steel-fibre contents may alter local heat conduction and reduce matrix homogeneity. Hybrid-fibre design therefore requires consideration of fibre type, content, aspect ratio, spatial distribution, and matrix compactness, rather than a simple comparison based on total fibre volume fraction.

In addition to steel–PP systems, steel–glass hybrid fibres primarily exploit the combined crack-control capabilities of the two fibre types. Raza et al. [31] investigated RPC containing different steel-to-glass fibre ratios after elevated-temperature exposure and reported that variations in the fibre combination produced different effects on individual residual mechanical properties. Similarly, Kumari et al. [7] showed that steel–glass hybridisation can influence the damage evolution of self-compacting concrete subjected to both thermal cycling and elevated temperatures.

A key distinction should nevertheless be maintained between steel–glass and steel–PP hybrid systems. The former can primarily be interpreted as a multiscale crack-bridging system, whereas the latter combines two fundamentally different physical functions: mass-transport regulation and mechanical crack bridging. Consequently, the term “hybrid effect” should always be associated with a clearly defined performance objective, such as spalling resistance, residual strength, crack control, toughness retention, or a combination thereof, rather than being used as a general statement that hybrid fibres are inherently superior to single-fibre systems.

### 3.5. Key Factors Governing Fibre Performance at Elevated Temperatures

This subsection briefly introduces the fibre-specific mechanistic roles of the principal controlling factors, while their quantitative cross-study effects are synthesised more systematically in Section 6.

#### 3.5.1. Fibre Content, Geometry, and Spatial Distribution

Fibre content is among the most frequently investigated variables, but it does not independently determine high-temperature performance. For steel fibres, increasing the volume fraction generally increases the probability that developing cracks will be bridged. Beyond a certain content, however, reduced workability, fibre agglomeration, and non-uniform orientation may offset the benefits associated with the additional fibres. This issue is even more important for PP fibres because their anti-spalling function depends strongly on whether fibre-induced interfaces, pores, and cracks form a sufficiently connected transport network [9,10].

The studies of Li et al. [9] and Zhang et al. [10] demonstrate that PP-fibre length, diameter, aspect ratio, number density, and three-dimensional spatial distribution can all affect the permeability and spalling behaviour of thermally exposed concrete. Consequently, a system with a relatively high fibre mass fraction but poor spatial distribution is not necessarily superior to one containing a lower fibre fraction with a more effective interconnected network.

#### 3.5.2. Fibre–Matrix Interface and Matrix Compactness

The role of fibres cannot be considered independently of the surrounding matrix. The same steel fibre may exhibit substantially different anchorage and thermal degradation behaviour in normal-strength concrete, HSC, and UHPC. A denser matrix generally provides stronger fibre bonding at ambient temperature but may also experience more pronounced brittle cracking and interfacial deterioration under severe thermal exposure [25,26].

For PP fibres, matrix compactness directly governs the importance of their transport-related function. In low-permeability HSC and UHPC, the formation of vapour-transport pathways by PP fibres can be particularly important, whereas the marginal benefit may be smaller in normal-strength concrete with relatively high initial permeability. Comparisons between studies are therefore meaningful only when differences in matrix strength, water-to-binder ratio, and pore structure are considered.

#### 3.5.3. Heating Rate, Maximum Temperature, and Thermal History

Fibre performance at elevated temperatures is strongly dependent on the thermal path. Rapid heating produces steeper temperature gradients and faster pore-pressure development, making the timing of transport-network formation by PP fibres particularly important. Slower heating, in contrast, may allow more time for internal moisture migration before critical pressure levels develop. The maximum exposure temperature further controls the extent of matrix degradation, steel fibre–matrix bond deterioration, and the residual effectiveness of glass fibres.

Thermal history must also be considered independently of peak temperature. A single exposure to 600 °C and repeated thermal cycling to 200 °C represent fundamentally different damage histories, even though the latter involves a much lower maximum temperature. Studies on steel–glass hybrid concrete under repeated thermal cycles have demonstrated that peak temperature alone is insufficient to characterise the cumulative thermal history of fibre-reinforced concrete [7].

#### 3.5.4. Moisture Condition and Specimen Scale

Explosive spalling is highly sensitive to the initial moisture content of concrete. Consequently, the anti-spalling effectiveness of PP or hybrid fibres should always be interpreted together with the moisture condition of the specimens. The same fibre system may perform very differently in a small, pre-dried laboratory specimen and in a large structural member with a high initial moisture content.

The distinction between material-scale specimens and structural members is equally important. Small cubes or cylinders generally provide shorter moisture-transport distances, whereas beams, slabs, and columns are additionally influenced by thermal gradients, restraint stresses, and externally applied loads. Fire tests on HSC columns by Khaliq and Kodur [32] demonstrated that the structural-level effectiveness of fibres ultimately reflects the interaction among material degradation, spalling, mechanical loading, and boundary restraint.

#### 3.5.5. Hot-State and Residual Properties

A further distinction required when interpreting the literature is that between hot-state properties and residual properties after cooling. Hot-state properties describe the mechanical response of concrete tested directly at the target temperature, whereas residual properties refer to the state of the material after completion of heating, thermal exposure, and cooling. Cooling can induce additional shrinkage, crack propagation, and interfacial changes, and the two types of data should therefore not be directly combined.

Khaliq and Kodur [28], for example, directly measured the thermal and mechanical properties of fibre-reinforced self-compacting concrete over the temperature range of 20–800 °C, whereas many other studies employed post-heating tests after the specimens had cooled. A reported “strength at 600 °C” can therefore represent substantially different physical states depending on the experimental protocol. Subsequent comparisons across studies should explicitly distinguish between these testing conditions.

Table 1 further demonstrates that different fibre systems have been investigated with substantially different performance objectives. Studies on steel fibres have focused predominantly on fibre–matrix bond, pull-out behaviour, and post-cracking performance; research on PP fibres has largely concentrated on pore structure, permeability, pore pressure, and spalling; studies on glass fibres have mainly examined residual mechanical behaviour; and investigations of hybrid fibres are increasingly moving beyond simple residual-strength comparisons towards mechanistic interpretation of interactions among different fibre types. This divergence in research emphasis itself demonstrates that the performance of different fibres should not be assessed using a single indicator such as residual compressive strength.

Overall, the high-temperature functions of fibres in concrete can be classified into three principal physical roles. First, steel fibres and, within a certain temperature range, glass fibres primarily provide crack bridging and stress transfer, thereby restricting the rapid opening and coalescence of thermally induced cracks. Second, PP fibres primarily provide permeability modification and pore-pressure relief by enhancing the connectivity of pores and cracks and thereby reducing the risk of explosive spalling. Third, hybrid fibres combine these mechanisms and can therefore influence multiple stages of thermal damage, from moisture transport and crack initiation to post-cracking load transfer.

Accordingly, the high-temperature performance of fibre-reinforced concrete should not be evaluated solely in terms of the extent to which fibre addition increases residual strength. A more rigorous assessment should identify the temperature range over which a fibre remains effective, the specific damage mechanism that it modifies, the conditions under which its contribution begins to deteriorate, and the extent to which its effectiveness is governed by fibre geometry and content, interfacial condition, matrix compactness, and thermal exposure regime. On the basis of these mechanistic considerations, the following section compares the macroscopic mechanical performance of steel-, PP-, glass-, and hybrid-fibre-reinforced concretes after exposure to elevated temperatures, with particular emphasis on compressive strength, tensile strength, flexural performance, elastic modulus, and toughness.

A similar distinction applies to permeability measurements. Hot or in situ permeability measured during heating reflects the transport capacity of concrete under the temperature conditions relevant to pore-pressure development and spalling, whereas post-heating or residual permeability characterises the pore–crack network remaining after thermal exposure and, where applicable, cooling. Residual permeability therefore provides important evidence of thermally induced network development but should not be interpreted as a direct measurement of vapour-transport capacity at the instant of spalling.

## 4. Residual Mechanical Properties of Fibre-Reinforced Concrete After Elevated-Temperature Exposure

Residual mechanical properties after elevated-temperature exposure provide an important basis for assessing the degree of fire-induced damage in fibre-reinforced concrete (FRC) and its remaining load-bearing capacity. As exposure temperature increases, dehydration of the cementitious matrix, deterioration of the interfacial transition zone, pore coarsening, and propagation of thermally induced cracks collectively lead to progressive reductions in compressive strength, tensile strength, flexural strength, elastic modulus, and toughness. These properties, however, do not exhibit the same sensitivity to thermal damage or fibre reinforcement. Existing experimental evidence indicates that residual compressive strength is governed more strongly by degradation of the cementitious matrix itself, whereas tensile, flexural, and post-cracking toughness responses are more closely associated with fibre bridging and fibre–matrix interfacial bonding [27,28,33,34].

To reduce the influence of differences in initial concrete strength when comparing results across studies, post-heating mechanical properties can be normalised using residual performance ratios. Representative expressions are given below.

*R_fc_*(*T*) = *f_c_*(*T*)/*f_c_*(20) (1)

*R_ft_*(*T*) = *f_t_*(*T*)/*f_t_*(20) (2)

*R_E_*(*T*) = *E*(*T*)/*E*(20) (3)

In Equations (1)–(3), *f_c_*(*T*), *f_t_*(*T*), and *E*(*T*) denote the post-cooling residual compressive strength, tensile strength, and elastic modulus, respectively, after exposure to temperature (*T*), while the corresponding denominators denote the reference properties at 20 °C. Hot-state quantities measured directly at the target temperature are distinguished separately in the text and are not pooled with these post-cooling residual ratios. Flexural strength, fracture energy, and toughness can be normalised in a similar manner. It is important to distinguish between hot-state testing and residual testing after cooling, because these protocols represent different material states and should not be combined indiscriminately. For example, Khaliq and Kodur [28] directly measured the strength and elastic modulus of heated material over the range 20–800 °C, whereas many other studies employed post-heating residual tests after cooling.

**Figure 3:**
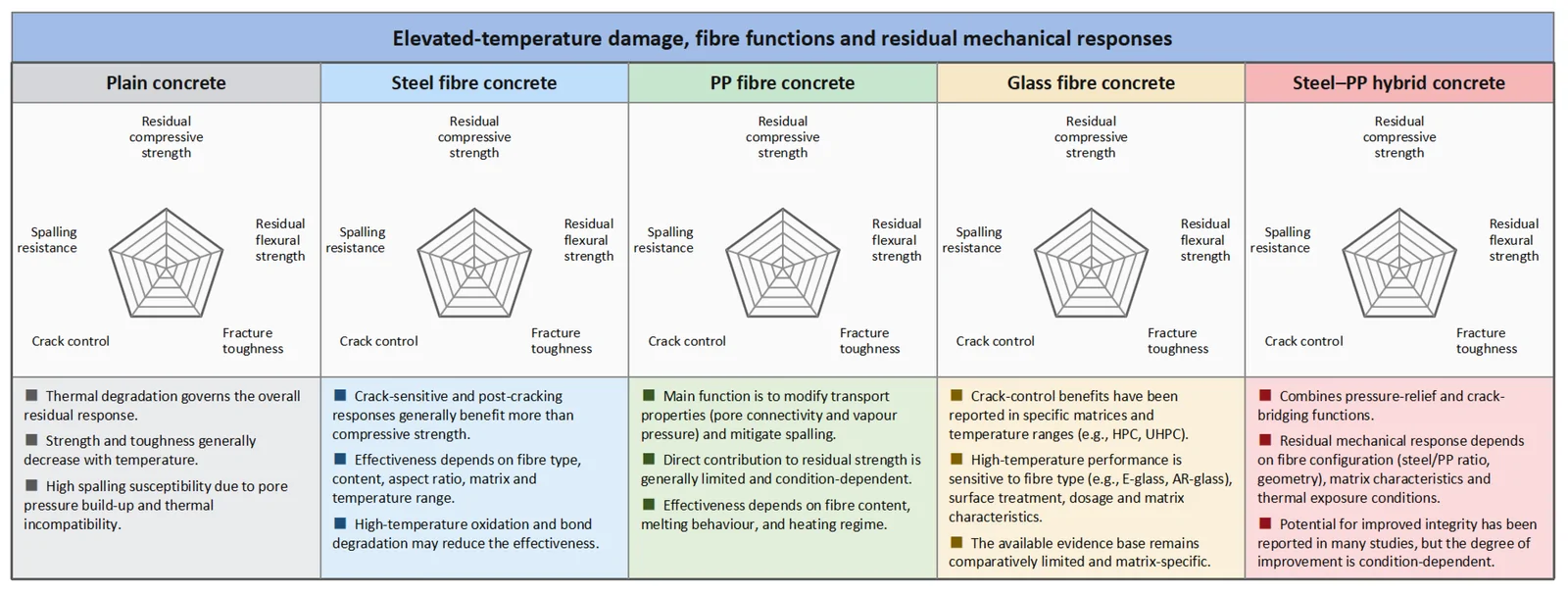
Relationship between elevated-temperature damage, fibre functions, and residual mechanical responses of fibre-reinforced concrete. The glass-fibre trends shown are qualitative and should be interpreted in view of the comparatively limited and matrix-specific evidence base.

The relationship between thermally induced damage, the dominant functions of different fibres, and the resulting residual mechanical responses is schematically summarised in Figure 3. The figure is intended as a qualitative mechanism-based synthesis and does not represent a quantitative ranking of fibre systems.

### 4.1. Residual Compressive Performance

Compressive strength is one of the most widely used indicators for assessing the extent of fire-induced damage in concrete. In general, the residual compressive strength of FRC ultimately decreases with increasing temperature, although the evolution is often stage-dependent. At low to intermediate temperatures, some studies have reported stable or even temporarily increased compressive strength. With further heating, decomposition of hydration products, aggregate–paste interfacial damage, and the development of interconnected cracking become dominant, resulting in pronounced strength loss. Consequently, local increases in compressive strength over a limited temperature interval should not be interpreted as evidence of a universal thermal-strengthening effect but should instead be evaluated in relation to matrix composition, heating regime, and testing protocol [33,34]. Zheng et al., for example, observed an increase in the residual compressive strength of steel-fibre-reinforced RPC at approximately 200–300 °C, followed by substantial degradation at higher temperatures over the overall range of 20–800 °C.

The contribution of steel fibres to compressive performance is mainly indirect. Under compression, concrete develops both axial and transverse cracking. Steel fibres crossing these cracks can restrain rapid crack propagation and mitigate localised material detachment, thereby helping the heated material retain greater overall integrity. Nevertheless, the peak compressive load remains governed primarily by the cementitious matrix and aggregate skeleton. Increasing the steel-fibre content therefore does not enhance residual compressive strength as directly as it enhances tensile or post-cracking performance [33,34].

As temperature increases further, degradation of the steel fibre–matrix bond and deterioration of the high-temperature mechanical properties of the fibres themselves progressively weaken this restraint. Steel-fibre geometry also affects residual performance. Guler and Akbulut compared concretes reinforced with 3D, 4D, and 5D steel fibres after exposure to 300, 500, and 800 °C and showed that increasing temperature substantially reduced residual strength and toughness, while the anchorage configuration at the fibre ends affected the extent of performance retention [27]. Recent studies on UHPC likewise indicate that the role of steel fibres cannot be assumed to remain uniformly beneficial under severe thermal exposure, because extensive matrix damage and fibre oxidation may reduce their relative reinforcing efficiency [35].

The influence of PP fibres on residual compressive strength is more complex. Their primary high-temperature function is to facilitate vapour escape and reduce the risk of explosive spalling rather than to continue carrying mechanical load after cooling. Fibre softening and melting promote the formation of transport pathways but also leave additional pores and interfacial defects. PP fibres may therefore preserve specimen integrity by preventing severe spalling without necessarily increasing the residual compressive strength of the cooled material [36,37]. Behnood and Ghandehari showed that PP fibres modify both compressive and splitting tensile responses of HSC after heating, with the magnitude and direction of the effect varying with temperature. Eidan et al. further demonstrated that PP-fibre length and content influence residual performance, while also showing that the fibres cannot prevent the severe loss of elastic modulus after exposure to 600 °C.

This distinction is important because spalling resistance and residual compressive-strength enhancement represent different performance objectives. Good resistance to explosive spalling does not necessarily imply a higher residual compressive strength of the remaining material.

The effect of glass fibres on compressive performance is likewise mainly associated with crack control. Wang et al. investigated concretes containing 0.60 and 2.54 cm glass fibres at different dosages after exposure to 440–1000 °C and showed that both fibre length and dosage influence residual compressive behaviour, although the effect is strongly dependent on temperature and fibre content [38]. It is therefore not valid to assume that increasing the glass-fibre dosage will continuously improve high-temperature compressive performance.

In steel–PP hybrid systems, PP fibres can reduce large-scale material loss caused by spalling, while steel fibres continue to restrain crack propagation. The two fibre types can therefore provide a more complete protection pathway. Chen and Liu showed that the combination of steel and PP fibres modified the residual compressive and splitting tensile performance of HSC over 20–800 °C, while emphasising that the anti-spalling function of PP fibres differs fundamentally from the mechanical reinforcement provided by steel fibres [23].

### 4.2. Residual Tensile and Flexural Performance

Compared with compressive behaviour, tensile and flexural properties are generally more sensitive to thermally induced cracking. Tensile failure is directly governed by crack initiation and coalescence, and interfacial or thermal cracks generated during heating may substantially reduce resistance to tensile failure even before a severe reduction in compressive load-bearing capacity occurs. The experiments of Behnood and Ghandehari similarly showed that splitting tensile strength is more sensitive to elevated-temperature exposure than compressive strength [37].

Steel fibres play a more direct role in this class of response. Once matrix cracking occurs, fibres bridging the cracks can continue to transfer tensile stress and delay crack localisation. Their contribution to residual tensile behaviour is therefore reflected not only in the peak load but also in a more gradual post-peak reduction in load-carrying capacity. Zheng et al. showed that steel-fibre content, fibre scale, holding time, and exposure temperature all influence the compressive and tensile responses of RPC after heating [34].

Flexural performance is particularly effective for revealing fibre-bridging action. Unlike plain concrete, in which load capacity decreases rapidly after first cracking, steel fibres can continue to transfer tensile stress across an opening crack. Consequently, residual flexural tensile strength after elevated-temperature exposure is generally more informative than peak flexural load alone. Gondokusumo et al. conducted tests on normal-weight concrete and lightweight steel-fibre-reinforced concrete over 200–800 °C and systematically evaluated residual flexural tensile strength, showing pronounced deterioration with increasing temperature [39].

For this reason, studies of steel-fibre-reinforced concrete should, where possible, report not only the maximum flexural strength but also residual flexural strengths corresponding to specified crack-mouth-opening-displacement (CMOD) levels. This distinction allows the effects of fibres on first-crack resistance and post-cracking load retention to be evaluated separately.

The role of PP fibres in tensile and flexural performance differs from that of steel fibres. Before severe softening occurs, PP fibres may contribute to the restraint of microcrack development; after exposure to sufficiently high temperatures, however, they no longer provide stable post-cracking bridging. Their principal contribution to residual tensile and flexural performance is therefore often indirect, through mitigation of severe spalling and preservation of specimen integrity rather than sustained tensile stress transfer after heating [36,37].

Recent experiments examining different PP-fibre lengths and contents further indicate that fibre geometry affects both compressive and fracture behaviour after thermal exposure, reinforcing the conclusion that PP-fibre effectiveness cannot be assessed on the basis of volume fraction alone [40].

Glass fibres can similarly improve tensile and flexural behaviour within a certain temperature range through microcrack bridging. In contrast to PP fibres, glass fibres do not lose their physical fibre form at approximately 160–170 °C and can therefore continue to contribute to crack control at relatively low temperatures. With further heating, however, deterioration of the fibres themselves and of the fibre–matrix interface progressively reduces their reinforcing effect. The available literature on glass-fibre-reinforced concrete at elevated temperatures remains substantially less extensive than that for steel or PP fibres, and many studies focus on specialised cementitious matrices. A universal residual tensile relationship across concrete classes therefore cannot yet be established [38,41].

### 4.3. Elastic Modulus and Stress–Strain Response

Elastic modulus is particularly sensitive to thermal damage because it reflects the overall stiffness of the material rather than a local peak load. As microcrack density increases, interfacial stiffness deteriorates, and the pore structure coarsens, the initial slope of the stress–strain curve may decrease substantially even when the concrete retains a considerable proportion of its compressive strength.

Khaliq and Kodur systematically measured the compressive strength, tensile strength, and elastic modulus of fibre-reinforced high-performance self-compacting concrete over 20–800 °C and demonstrated a pronounced reduction in elastic modulus with increasing thermal damage [28]. Tests on PP-fibre-reinforced concrete likewise show that fibre incorporation cannot prevent the overall stiffness degradation caused by elevated temperatures [36].

The influence of fibres on elastic modulus is generally weaker than their influence on post-cracking performance. At small strains, before the formation of pronounced macrocracks, fibre bridging is not yet fully mobilised. The modulus is governed mainly by the stiffness of the cementitious matrix, aggregates, and interfacial regions. Once these phases are severely damaged by heating, steel, PP, and glass fibres alike are unable to prevent substantial stiffness loss.

Elevated temperatures also alter the complete stress–strain response. Typical changes include a reduction in the initial slope, a decrease in peak stress, an increase in peak strain, modification of the post-peak descending branch, and increased nonlinear deformation during failure. Post-fire constitutive assessment should therefore not rely solely on a residual compressive-strength factor. For structural analysis, the residual elastic-modulus ratio defined in Equation (3), together with the peak-strain ratio defined below, should be considered.

*R_εp_*(*T*) = *ε_p_*_,*T*_/*ε_p_*_,20_ (4)

The principal advantage of steel fibres appears in the nonlinear and post-peak portions of the stress–strain response. Once macrocracking develops in the matrix, steel fibres can maintain part of the load transfer across cracks and thereby reduce the rate of post-peak strength loss. A comparison based only on peak strength may therefore underestimate the actual mechanical value of steel fibres after elevated-temperature exposure.

### 4.4. Toughness and Fracture Performance

Toughness and fracture performance are among the most important indicators of the high-temperature behaviour of FRC, yet they are often overshadowed by compressive strength. The principal advantage of fibre reinforcement is not merely to increase the maximum load reached before failure, but to continue transferring stress, dissipating energy, and delaying crack instability after matrix cracking. From the standpoint of fibre functionality, fracture energy and residual toughness can therefore provide a more direct representation of fibre reinforcement than peak compressive strength.

For steel fibres, post-heating toughness is governed by the remaining mechanical properties of the fibres, fibre–matrix bond retention, and geometric anchorage. After crack formation, fibre pull-out, sliding, and plastic deformation dissipate energy, producing a post-peak response markedly different from that of plain concrete.

Guler and Akbulut [27] showed that the residual strength and toughness of concretes reinforced with 3D, 4D, and 5D steel fibres all decreased substantially with increasing temperature, while pronounced differences remained among anchorage configurations. This indicates that post-heating toughness depends not simply on the presence of steel fibres but also on fibre geometry and the ability of the interface to transfer load.

More recent studies have evaluated heated steel-fibre-reinforced concrete using combined measures of compressive strength, peak flexural load, equivalent flexural strength, and toughness, and have proposed toughness-based assessment approaches for thermally damaged SFRC. This development reflects a broader shift in elevated-temperature FRC research from peak-strength metrics towards complete load–deformation behaviour and energy-absorption capacity [42].

PP fibres exhibit a dual influence on fracture behaviour. At relatively low temperatures, they can restrain microcracking, while at higher temperatures the transport channels formed after melting can reduce severe spalling. Once melted, however, the fibres are no longer able to provide substantial post-cracking bridging. Residual fracture performance therefore reflects competition between reduced spalling damage and the loss of fibre bridging capacity [36,40].

Steel–PP hybrid systems demonstrate the functional complementarity of these two mechanisms. PP fibres primarily reduce pore-pressure accumulation and spalling risk during heating, whereas steel fibres continue to carry post-cracking load once cracking develops. Ding et al. evaluated self-compacting high-performance concrete reinforced with steel fibres, PP fibres, and a steel–PP fibre cocktail using residual compressive strength, ultimate load, fracture energy, and flexural toughness. Their results indicated that PP microfibres alone provided only a limited direct contribution to residual mechanical performance, whereas the combination with high-melting load-bearing fibres produced a more balanced high-temperature response [43].

Accordingly, toughness assessment of FRC after elevated-temperature exposure should place particular emphasis on the following response measures:

Residual flexural strength;
Load–deflection response;
Load–CMOD response;
Fracture energy;
Flexural toughness;
Post-cracking load-carrying capacity.

These indicators provide a more reliable basis for distinguishing between two different material-design objectives: retention of peak strength and improvement of damage tolerance.

### 4.5. Integrated Comparison of Different Fibre Systems

Table 2 summarises representative original experimental studies on FRC subjected to elevated temperatures. The studies differ substantially in matrix type, fibre dosage, fibre geometry, temperature level, holding time, and cooling regime. They are therefore more appropriate for identifying trends and mechanisms than for constructing a direct ranking from absolute strength values reported across different publications. To avoid conflating different material states, the testing state and cooling regime are explicitly identified in the comparative tables wherever this information is available from the original studies. Hot-state measurements and residual properties obtained after cooling are treated as separate categories.

Taken together, these experiments reveal an important point: no single fibre type maintains a universal advantage across all residual mechanical properties. Steel fibres act most directly on crack bridging, tensile and flexural behaviour, and toughness. The principal value of PP fibres lies in mitigating catastrophic material loss caused by spalling, while their direct contribution to residual material strength remains strongly condition-dependent. Glass fibres provide a degree of crack control, but the current high-temperature database is comparatively limited in both volume and material coverage. Hybrid systems derive their principal advantage from the functional complementarity of different fibre types (Table 3).

On this basis, the mechanical indicators can be further classified according to their sensitivity to fibre action. Compressive strength is primarily a matrix-dominated property: fibres can modify the failure process but cannot eliminate thermal decomposition of the cementitious matrix. Tensile and flexural strengths are crack-dominated properties and are therefore more sensitive to steel fibres and other fibres capable of retaining bridging capacity at elevated temperatures. Elastic modulus is an overall-damage-dominated property and generally decreases rapidly with increasing microcracking and interfacial deterioration. Toughness and fracture energy, in contrast, are strongly fibre-dominated properties and most directly reflect fibre pull-out, bridging, and post-cracking energy dissipation. This classification is broadly consistent with experimental observations for steel-, PP-, and hybrid-fibre systems.

Residual compressive strength alone is therefore insufficient for evaluating the post-fire performance of FRC. A more appropriate minimum set of material-level indicators should include compressive strength, tensile or flexural strength, elastic modulus, and fracture energy or toughness, representing load-bearing capacity, crack-sensitive behaviour, overall stiffness, and post-cracking damage tolerance, respectively.

For steel-fibre and hybrid-fibre systems, complete load–displacement or load–CMOD curves should be retained whenever possible, because peak-strength comparisons alone can substantially underestimate the contribution of fibres to post-cracking performance.

Overall, increasing temperature ultimately degrades the mechanical performance of all FRC systems. Fibres do not eliminate thermal damage; rather, they alter the damage pathway and the manner in which residual load is carried. Steel fibres primarily preserve tensile, flexural, and toughness performance through crack bridging; PP fibres mainly preserve material integrity indirectly by mitigating severe spalling; glass fibres can contribute to crack control within a limited temperature range; and hybrid fibres combine transport regulation with post-cracking load transfer to provide greater adaptability across multiple stages of thermal damage. Building on these observations, the next section examines the effects of different fibre systems on high-temperature cracking, surface scaling, and explosive spalling behaviour.

## 5. Cracking and Spalling Behaviour of Fibre-Reinforced Concrete at Elevated Temperatures

High-temperature material behaviour, fire-induced spalling, and structural fire performance represent different levels of assessment and should not be used interchangeably. In this review, material behaviour refers primarily to specimen-scale changes in mechanical, fracture, and transport properties under prescribed thermal exposure; fire-induced spalling refers to the thermally induced detachment or explosive loss of concrete during heating; and structural fire performance refers to the response of structural members under combined thermal gradients, mechanical loading, reinforcement restraint, and boundary conditions. Accordingly, material-scale behaviour or spalling resistance should not be interpreted directly as evidence of structural fire resistance unless supported by member-scale fire tests. Thermal cracking generally develops progressively as a consequence of cement-matrix dehydration, thermally incompatible deformation between aggregates and the surrounding paste, and temperature gradients, evolving from isolated microcracks into interconnected macrocracks. Spalling, in contrast, refers to the sudden detachment of surface or localised concrete during heating and may, in severe cases, develop into explosive loss of material. For high-strength concrete (HSC), high-performance concrete (HPC), and ultra-high-performance concrete (UHPC), dense matrices improve mechanical performance and durability at ambient temperature but simultaneously restrict internal vapour transport. Consequently, under certain moisture conditions and heating regimes, these materials may exhibit a greater susceptibility to severe spalling [3,6,24,44].

**Figure 4:**
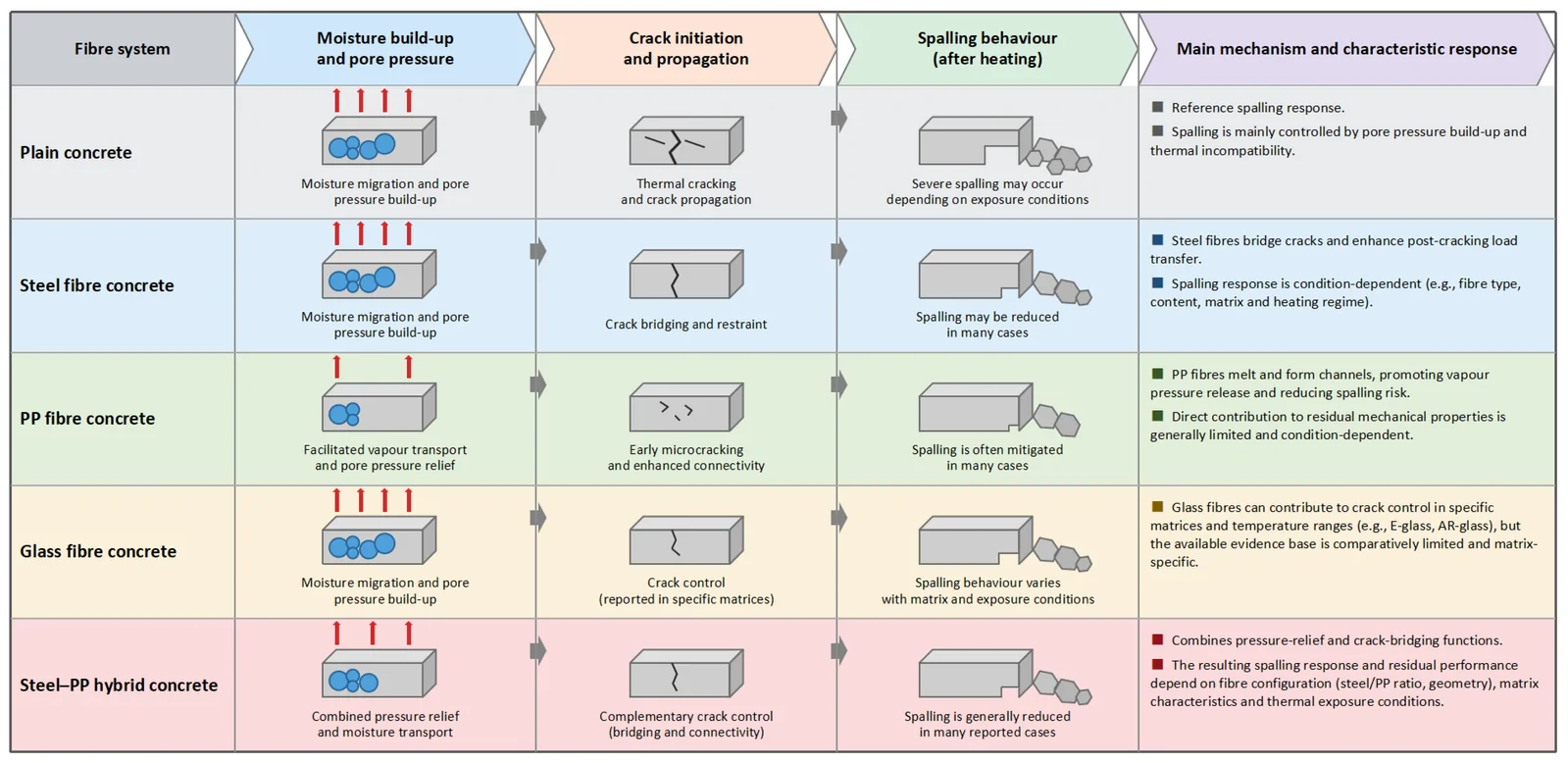
Fibre-dependent pathways governing cracking, vapour transport, and spalling of concrete exposed to elevated temperatures.

Fibres do not mitigate these two forms of damage through a common mechanism. Steel fibres primarily restrain crack opening and coalescence but do not necessarily ensure a reduction in pore-pressure accumulation. Polypropylene (PP) fibres mainly reduce spalling risk by modifying pore connectivity and permeability after heating. Glass fibres act predominantly through crack bridging, while the current evidence supporting their anti-spalling effectiveness remains substantially less extensive than that available for PP fibres. Steel–PP hybrid systems can simultaneously influence vapour transport and crack propagation. Accordingly, rather than reiterating the fundamental physicochemical origins of thermal damage, this section focuses on how different fibre systems modify the interaction among crack development, vapour transport, and spalling, as schematically illustrated in Figure 4 [11,30,45,46,47]. The comparisons shown in Figure 4 therefore indicate fibre-dependent mechanisms and conditional tendencies rather than universal differences in spalling resistance.

### 5.1. Fundamental Manifestations of Thermal Cracking and Spalling

Cracking in heated concrete generally develops from dispersed microcracking towards localised macrocracking. At relatively low temperatures, cracks tend to initiate preferentially within the interfacial transition zone between aggregates and cement paste and around pre-existing defects. As thermal damage accumulates, crack density, crack width, and connectivity progressively increase, eventually producing interconnected crack networks. Such crack development has two simultaneous consequences. Mechanically, it reduces stiffness and tensile resistance; from a transport perspective, it creates additional pathways for water vapour and gas migration. Crack evolution therefore acts both as a manifestation of mechanical damage and as a mechanism of transport regulation [8,11]. SEM and MIP observations reported by Yermak et al. showed that steel fibres can reduce crack opening during heating, while such crack control also modifies the surrounding pore and gas-transport conditions.

At the macroscopic scale, heated concrete may exhibit surface cracking, localised surface detachment, corner spalling, or explosive spalling, depending on the severity of thermal damage. Explosive spalling is particularly hazardous because material may be suddenly ejected during heating, potentially exposing deeper concrete layers or reinforcement. The consequences extend beyond superficial material loss. A reduction in cover thickness accelerates heat penetration into the structural interior and may reduce both the effective load-bearing cross-section and the fire-resistance duration of the member. Tests on UHPC and HSC, including large-scale column fire tests, have demonstrated that spalling can become a critical factor governing structural fire performance [32,48].

It is particularly important not to equate “more cracking” directly with “more severe spalling”. From the perspective of structural integrity, extensive cracking clearly indicates greater damage. From the perspective of vapour transport, however, a sufficiently fine and interconnected microcrack network may enhance permeability and reduce internal pore-pressure accumulation. High-temperature FRC therefore involves a fundamental competition between crack restraint and permeability enhancement.

If crack opening is strongly restrained while no alternative vapour-release pathways are available, internal pressure may continue to accumulate. Conversely, if a sufficiently connected network of fine pores and microcracks develops, pressure may be released without the formation of large-scale destructive cracking. This competition provides a key mechanistic explanation for the different roles of steel and PP fibres and, in turn, for the potential advantages of hybrid-fibre systems [46,49].

### 5.2. Role of PP Fibres in Spalling Mitigation

Among the principal fibre types used in concrete, PP fibres exhibit the clearest mechanistic relationship with high-temperature spalling mitigation and are supported by the most extensive experimental evidence. Early work by Kalifa et al. on HPC demonstrated that PP fibres can substantially modify permeability and pore structure after heating, with pronounced changes emerging near the thermal range associated with PP-fibre softening and melting [3]. Numerous subsequent studies have confirmed that the principal function of PP fibres in spalling mitigation is not to continue carrying mechanical load but to enhance the ability of water vapour to migrate through the heated concrete.

This effect should be understood as a progressive transport-network development process. During heating, thermal mismatch and interfacial damage can develop around PP fibres before complete melting. Subsequent fibre softening and melting further enlarge or connect these interfacial regions, facilitating the formation of an interconnected network of pores and microcracks. As permeability increases, vapour can migrate more readily through the matrix, limiting pore-pressure accumulation and thereby reducing the likelihood of explosive spalling.

Importantly, this mechanism should not be oversimplified as the assumption that PP fibres melt at approximately 160–170 °C and leave behind empty cylindrical channels identical to the original fibre geometry. Interfacial damage, thermal-expansion mismatch, fibre softening, and final melting all contribute to the development of the transport network. Zeiml et al., through in situ concrete fire testing, showed that increasing PP-fibre content could reduce spalling depth and related this response to the associated increase in permeability [6].

The geometry and spatial distribution of PP fibres are equally important. If the voids remaining after individual fibres soften or melt remain isolated, their contribution to global vapour transport will be limited. Effective pressure relief requires these fibre-related voids to become interconnected through interfacial microcracks and the surrounding pore network. This explains why PP fibres with the same mass dosage can exhibit markedly different anti-spalling performance depending on fibre length, diameter, aspect ratio, number density, and spatial distribution.

Ozawa and Morimoto compared different organic fibres and demonstrated that fibre type and thermal behaviour strongly influence pore-pressure development and spalling in HPC [44]. Their results further indicate that anti-spalling performance is closely associated with the formation of pressure-release pathways as fibres soften, dissolve, or melt.

PP-fibre dosage also exhibits an effective range rather than a simple monotonic relationship with fire performance. Insufficient fibre content may fail to establish a sufficiently connected transport network, whereas excessive fibre content may reduce fresh-state workability and leave additional porosity after cooling, potentially impairing residual mechanical properties. The design objective for PP fibres should therefore not be expressed as “more fibres provide better fire resistance”. A more rational concept is to establish the minimum effective fibre network required to provide sufficient vapour transport and pressure relief.

This issue is particularly important in UHPC because of its extremely low initial permeability. In such dense systems, whether PP fibres can generate a sufficiently connected transport network may directly influence whether explosive spalling occurs. A series of UHPC studies by Li et al. demonstrated that PP fibres, aggregate size, and steel fibres can all influence pore pressure and spalling through their effects on the high-temperature permeability network, indicating that the role of PP fibres cannot be assessed independently of the surrounding crack and pore structure [46].

### 5.3. Effects of Steel and Glass Fibres on Cracking and Spalling

The primary function of steel fibres at elevated temperatures differs fundamentally from that of PP fibres. Steel fibres bridging developing cracks can reduce crack-opening width and delay the transition from microcracking to macrocracking. This generally benefits overall integrity, limits local material detachment, and enhances post-cracking load transfer. From the perspective of spalling mitigation, however, the role of steel fibres is not invariably beneficial [11,45].

Yermak et al. showed that steel fibres effectively restrained thermal crack opening, whereas PP fibres were more effective in preventing macroscopic damage under ISO fire exposure [11]. Algourdin et al. further demonstrated that steel-fibre-reinforced concrete could still experience spalling under different heating regimes, while the addition of a relatively small amount of PP fibre suppressed spalling in their hybrid mixtures [45]. These findings emphasise that crack control is not equivalent to pore-pressure relief.

The reason is that reducing crack opening can improve structural integrity while simultaneously limiting vapour-release pathways that might otherwise be created by wider cracks. At the same time, thermal-expansion mismatch between steel fibres and the surrounding matrix may generate fine interfacial cracks that increase local permeability. The net effect of steel fibres on spalling therefore reflects competition between two opposing mechanisms: macrocrack restraint and microcrack-assisted permeability enhancement.

This competition helps explain why different studies have reported different spalling responses for steel-fibre-reinforced concrete. The outcome is further affected by fibre content, fibre geometry, matrix strength, initial moisture condition, and heating rate [11,45].

Glass fibres likewise act predominantly through crack bridging and crack refinement rather than through the formation of melt-induced vapour-release pathways. Raza et al. reported that 2–4 vol.% glass fibres reduced spalling in the investigated RPC system and that fibre content influenced residual behaviour after exposure to 200–800 °C [30].

These findings, however, should not be generalised without qualification. The experimental database on glass-fibre-related spalling remains substantially smaller than that available for PP and steel fibres, and much of the existing evidence is derived from highly dense matrices such as RPC. The current literature therefore supports the view that glass fibres possess crack-control capability with potential spalling-mitigation effects but does not yet justify treating them as a universally reliable anti-spalling fibre comparable to PP fibres.

### 5.4. Complementary Effects of Hybrid Fibres on Spalling Mitigation

Steel–PP hybrid reinforcement is one of the most mechanistically coherent fibre combinations investigated for high-temperature concrete. PP fibres primarily address vapour migration and pore-pressure accumulation during heating but lose their capacity for sustained post-cracking load transfer after softening and melting. Steel fibres, in contrast, remain mechanically active and preserve post-cracking integrity, but do not necessarily provide sufficient pressure relief when used alone. Their combination can therefore simultaneously target pressure relief and crack control.

The steel–PP concept should not be interpreted simply as the arithmetic addition of the effects of two fibre types. Rather, it represents a division of functions across different stages of thermal damage. Pliya et al. investigated steel- and PP-fibre cocktails in HSC and showed that hybrid systems can improve the overall high-temperature response because the two fibres provide different functional contributions [24]. Bangi and Horiguchi subsequently measured internal pore pressure at different depths in HSC and hybrid-fibre HSC, demonstrating that hybrid reinforcement can modify pore-pressure development and that heating rate substantially affects the measured pressure response [47].

More direct evidence of mechanistic synergy was provided by Li et al. in UHPC. Their observations showed that PP–steel hybrid systems could prevent explosive spalling at relatively low fibre contents and that voids or tunnels generated after PP-fibre melting could become interconnected through thermally induced microcracks associated with both steel and PP fibres [8]. This enhanced network connectivity substantially increased permeability and reduced pore-pressure accumulation.

At the same time, steel fibres continued to bridge cracks and restrain macrocrack opening, thereby helping preserve residual material integrity. This interpretation is more physically rigorous than the simplified statement that “PP fibres prevent spalling while steel fibres improve strength”, because steel fibres may also participate indirectly in transport-network development through thermally induced interfacial cracking [8].

Subsequent work by Li et al. investigating the combined effects of aggregate size, steel fibres, and PP fibres on UHPC spalling and pore pressure further confirmed that spalling mitigation depends on the overall microcrack–pore network rather than on a single material parameter [46]. In another study, Li and Zhang considered lateral restraint and showed that external confinement alters thermal stress, pore-pressure development, and spalling behaviour. Thus, even for an identical fibre composition, different mechanical boundary conditions may produce different spalling outcomes [48].

The potential structural relevance of these material-scale interactions has also been examined in member-scale fire tests. Khaliq and Kodur compared HSC columns containing no fibres, PP fibres, steel fibres, and hybrid fibres under fire exposure and found that hybrid-fibre HSC columns provided the best overall fire response among the tested systems under their experimental conditions [32].

Hybrid reinforcement should nevertheless not be interpreted as implying that higher total fibre contents are always beneficial. Excessive PP and steel fibre contents may reduce workability, impair fibre distribution, and alter the initial pore structure. The optimum fibre combination also depends on water-to-binder ratio, aggregate size, heating rate, specimen dimensions, and restraint conditions. A more useful research objective is therefore to establish quantitative relationships among matrix characteristics, fibre-network architecture, thermal loading, and spalling response, rather than to search for a universal optimum dosage independent of material and testing conditions.

### 5.5. Key Factors Governing Spalling-Mitigation Effectiveness

The following discussion is limited to the spalling-specific implications of these factors; their broader quantitative influence on high-temperature FRC performance is integrated in Section 6.

#### 5.5.1. Matrix Strength and Initial Permeability

Matrix compactness governs the relative importance of fibre-generated transport pathways for spalling mitigation. Dense HSC and UHPC generally depend more strongly on PP-fibre-induced permeability enhancement than normal-strength concrete with higher initial permeability [8,46].

Consequently, an identical PP-fibre dosage does not have the same physical significance in normal-strength concrete and UHPC.

#### 5.5.2. Initial Moisture Content

Initial moisture condition controls the available vapour source and should therefore be considered together with permeability and heating rate when comparing fibre-related spalling resistance.

#### 5.5.3. Heating Rate and Fire Exposure Regime

Heating rate governs the rate of vapour generation and the development of thermal gradients; consequently, identical peak temperatures do not necessarily represent equivalent spalling conditions [45,47].

Algourdin et al. further observed that plain concrete could exhibit different spalling behaviour under different heating programmes and that steel-fibre systems were also sensitive to the heating regime [45]. Thus, exposure to the same maximum temperature, for example 600 °C, does not necessarily imply that specimens experienced the same thermal-damage history.

#### 5.5.4. Fibre Geometry and Spatial Connectivity

For PP fibres, fibre length, diameter, aspect ratio, and number density govern the number and spacing of potential transport pathways within a unit volume of concrete. For steel fibres, geometry and orientation influence both crack bridging and thermally induced interfacial microcracking. High-temperature design therefore cannot be reduced to fibre volume fraction alone.

From the perspective of spalling control, a more complete description should consider fibre volume fraction, fibre length, fibre diameter, aspect ratio, fibre number density, and transport-network connectivity. Fibre number density refers to the number of fibres per unit volume, while transport-network connectivity is used here only as a conceptual descriptor of the degree to which fibre-related pores and cracks form interconnected transport pathways, rather than as a presently standardised engineering parameter.

#### 5.5.5. Specimen Scale and Restraint Conditions

Small laboratory specimens provide relatively short vapour-transport distances, whereas full-scale walls, slabs, beams, and columns exhibit substantially longer transport paths and are additionally influenced by reinforcement, externally applied load, and mechanical restraint. Therefore, the absence of spalling in small specimens cannot automatically be interpreted as evidence of sufficient fire safety at the structural scale.

Tests on restrained UHPC by Li and Zhang and on large-scale HSC columns by Khaliq and Kodur both demonstrate that restraint conditions and structural scale can substantially modify the contribution of fibres to spalling and overall fire resistance [32,48].

### 5.6. Evaluation Metrics and Cross-Study Comparison of High-Temperature Spalling

Many studies use a simple “spalling/no-spalling” classification as the primary outcome. Although useful as a first-level indicator, such binary classification cannot distinguish minor surface detachment from severe explosive cross-sectional loss. A more comprehensive evaluation of spalling resistance in fibre-reinforced concrete should, where possible, report spalling occurrence, onset temperature or onset time, maximum spalling depth, spalled area, spalled mass or mass fraction, peak pore pressure, permeability evolution, maximum crack width, and residual cross-sectional area. These recommendations are broadly consistent with the consensus guidance developed by RILEM TC 256-SPF. The RILEM recommendations address material screening tests for fire-spalling propensity and provide complementary guidance for observing and quantifying spalling during standardised fire-resistance tests [50,51]. In the context of fibre-reinforced concrete, the present review additionally emphasises recording fibre type and geometry, moisture condition, thermal history, pore-pressure and permeability evolution, and residual mechanical response so that spalling severity can be interpreted together with the underlying transport and crack-control mechanisms.

These parameters describe different dimensions of the phenomenon, including whether spalling occurs, when it begins, its severity, and the transport or mechanical processes associated with it. Accordingly, the term “spalling” is used here as a general descriptor only when the original study does not provide a more specific classification; where sufficient information is available, surface or localised spalling, material loss, and explosive spalling are distinguished, and quantitative indicators of onset and severity are prioritised over a simple binary classification.

For comparisons among fibre systems, reporting only whether spalling occurred is therefore insufficient. One fibre system may not entirely eliminate local surface spalling but may substantially reduce spalling depth, whereas another may suppress visible explosive spalling at the expense of increased internal porosity or reduced residual strength. The work of Sideris and Manita on fibre-reinforced self-compacting concrete provides a representative example: PP fibres were effective in suppressing explosive spalling, while residual mechanical performance could simultaneously be adversely affected [49].

Spalling resistance should therefore ultimately be evaluated together with the residual mechanical properties discussed in the preceding section rather than as an independent performance criterion. To illustrate how different fibre systems have been assessed under varying material and thermal conditions, representative experimental studies on cracking and spalling are summarised in Table 4, with quantitative indicators of spalling severity retained where they are reported in the original studies. The comparison highlights the strong dependence of spalling behaviour on fibre type, matrix characteristics, heating regime, moisture condition, and structural restraint.

Table 4 further demonstrates that the influence of fibres on spalling cannot be ranked independently of testing conditions. PP fibres show the most direct anti-spalling effect in many studies on dense concrete matrices, but their efficiency is governed by dosage, fibre geometry, and transport-network connectivity. Steel fibres primarily restrain crack opening, while their independent contribution to spalling mitigation is more strongly condition-dependent. Glass fibres show some potential for reducing cracking and surface loss, although the available evidence remains limited. Steel–PP hybrid systems currently exhibit the clearest mechanistic complementarity because they simultaneously influence vapour transport and post-cracking integrity.

Overall, fibre-induced control of cracking and spalling at elevated temperatures should not be interpreted simply as a reduction in the number of cracks. The critical issue is the balance among crack scale, crack connectivity, vapour-transport capacity, and the residual fracture resistance of the material. Steel fibres preserve macroscopic integrity through crack bridging; PP fibres reduce pore pressure by promoting an effective transport network; glass fibres primarily restrain crack development; and steel–PP hybrid fibres can regulate both the macroscopic crack system and the mesoscopic permeability network. For engineering applications, the most rational fibre-design objective is therefore not to eliminate cracking completely, but to establish controlled and effective pressure-release pathways while maintaining sufficient post-cracking load-bearing capacity.

## 6. Key Factors Governing the High-Temperature Performance of Fibre-Reinforced Concrete

Building on the factor-specific discussions in Section 3.5 and Section 5.5, this section provides the principal integrated cross-study synthesis, with emphasis on quantitative evidence and transferable comparison rules. The preceding sections have demonstrated that steel, polypropylene (PP), glass, and hybrid fibres can modify the high-temperature response of concrete through crack bridging, vapour-transport regulation, and post-cracking stress transfer. However, cross-study comparisons indicate that fibre type alone cannot account for the substantial variability reported in the literature. The same fibre system may completely suppress spalling in one study, merely reduce its severity in another, or show little measurable benefit under different testing conditions. Similarly, pronounced scatter in residual mechanical properties can occur even when specimens are exposed to the same nominal peak temperature. Such discrepancies arise because the high-temperature response is jointly controlled by the thermal exposure path, fibre-network characteristics, matrix compactness, and initial moisture condition [3,6,45,47,52,53,54,55]. Accordingly, rather than treating peak temperature, heating rate, fibre length, moisture content, and related parameters as isolated variables, this section groups them into several interacting control levels and uses representative experimental evidence to illustrate how they govern the actual thermal response of fibre-reinforced concrete.

From a mechanistic perspective, the high-temperature behaviour of FRC can be interpreted as the combined outcome of three interacting processes. First, the thermal exposure path governs the rates of moisture evaporation, thermal-gradient development, and matrix degradation. Second, fibre number, dimensions, and spatial distribution determine whether an effective crack-bridging network or vapour-release network can develop. Third, the initial permeability and moisture state of the matrix determine both the amount of vapour that must be released and the resistance encountered during vapour transport. Only by considering these factors simultaneously can apparently contradictory observations across different studies be rationalised.

### 6.1. Thermal Exposure Path Determines the Actual Damage State

Peak temperature alone is not a sufficient descriptor of thermal exposure. Many studies classify specimens according to nominal temperatures such as 200, 400, 600, or 800 °C. However, identical furnace temperatures do not imply identical thermal histories. Heating rate controls the temperature difference between the surface and interior and the rate of vapour generation; holding duration determines whether the specimen interior approaches the target temperature; and the cooling regime may introduce additional thermal shock after heating. Consequently, direct comparison among studies based only on peak temperature is an important source of apparent scatter in the high-temperature concrete literature.

Algourdin et al. [45] provide a representative example. The authors compared plain concrete, concrete containing 60 kg/m^3^ steel fibres, and hybrid concrete containing 60 kg/m^3^ steel fibres plus 0.75 kg/m^3^ PP fibres under either constant heating at 10 °C/min or the ISO 834 standard [56] fire curve. The addition of 60 kg/m^3^ steel fibres alone did not consistently improve spalling resistance and, particularly under ISO 834 exposure, was associated with greater spalling susceptibility. In contrast, supplementing the steel-fibre system with only 0.75 kg/m^3^ PP fibres prevented spalling under both thermal-loading regimes. The study further showed that cylindrical specimens experienced more severe spalling than slab specimens. These observations demonstrate that whether steel fibres improve fire performance cannot be evaluated independently of heating regime and specimen thermal boundary conditions.

Choe et al. [52] further demonstrated the interaction between heating rate and matrix water-to-binder ratio (W/B). In high-strength concretes with different W/B values subjected to fast and slow heating, moisture migration and pore-pressure development varied as a function of both parameters. Under rapid heating, HSC with W/B below 0.33 exhibited surface spalling associated with near-surface moisture clogging. Rapid heating therefore not only increases thermal gradients but also shortens the time available for vapour migration, making dense matrices more likely to enter a condition in which vapour generation exceeds vapour-release capacity.

Cooling history exerts an equally important first-order influence on residual performance. Peng et al. [53] exposed fibre-reinforced concrete to temperatures between 200 and 800 °C and subsequently applied natural cooling, water spraying for 5–60 min, or direct water quenching. Compared with natural cooling, both spraying and quenching produced greater losses in compressive strength, splitting tensile strength, and fracture energy. When spraying duration reached approximately 30 min or longer, the resulting mechanical deterioration approached that caused by direct quenching. This finding indicates that laboratory tests based solely on natural cooling cannot be assumed to represent the post-fire condition of concrete subjected to rapid cooling by firefighting water.

High-temperature exposure should therefore be defined in terms of a thermal path rather than peak temperature alone. Cross-study datasets should, at minimum, record peak temperature, heating rate or fire curve, holding duration, and cooling regime. For spalling analysis in particular, low-rate furnace heating, rapid heating on the order of 10 °C/min, and standard fire curves such as ISO 834 should be distinguished because they produce fundamentally different pore-pressure and thermal-stress histories [7,45,47,52,53].

### 6.2. Fibre Effectiveness Is Governed by the Effective Network Rather than Dosage Alone

Fibre-related parameters are among the most readily adjustable design variables in high-temperature FRC, yet fibre mass or volume fraction alone provides only a coarse description of the system. For steel fibres, post-fire performance depends primarily on the probability that cracks are bridged, together with fibre–matrix bond and mechanical anchorage. For PP fibres, anti-spalling performance depends on whether fibre number density, length, diameter, and spatial distribution generate a sufficiently connected vapour-transport network. Consequently, identical fibre volume fractions do not necessarily produce equivalent high-temperature behaviour [8,9,29,46,55,57,58,59].

PP fibres exhibit a clear minimum-effective-network behaviour. Han et al. [54] investigated HPC with W/B values of 0.30 and 0.40 and PP-fibre volume fractions of 0, 0.05, and 0.10%. Severe spalling occurred in all specimens without PP fibres, whereas no spalling was observed when the PP content reached 0.05 vol.%—approximately 0.45 kg/m^3^ in that study—or higher. Specimens containing PP fibres alone retained approximately 70% of their original compressive strength, whereas the combination of PP fibres and lateral metallic fabric retained approximately 90%. Correspondingly, mass loss decreased from values approaching 60% in systems without PP fibres to below 10% in the fibre-containing systems. These findings clearly indicate that the primary benefit of PP fibres is the preservation of specimen integrity rather than a direct enhancement of residual compressive strength.

Li et al. [46] subjected UHPC specimens to one-sided heating at 2 °C/min and found that adding 3 kg/m^3^ PP fibres increased the residual intrinsic permeability from 0.733 × 10^−18^ to 13.8 × 10^−18^ m^2^, corresponding to an increase of approximately 19-fold. Increasing aggregate size alone produced only an approximately twofold increase. More importantly, the combination of PP fibres with larger aggregates increased the residual permeability by approximately 1500-fold relative to the reference mixture, while the PP–steel fibre combination produced an increase of approximately 1040-fold. The authors also observed a liquid-water vaporisation plateau at approximately 115–125 °C and a second temperature plateau beginning near 180 °C, the latter being associated with the activation of PP-fibre-related transport mechanisms. These results show that the advantage of hybrid systems cannot be interpreted as a simple superposition of fibre properties; rather, microcracks induced by differential thermal deformation can enhance the connectivity among PP-fibre-generated channels.

Fibre geometry also determines whether a transport network reaches a percolating state. Li et al. [9], based on 15 UHPC mixtures, showed that increasing PP-fibre length and dosage had a stronger effect on residual permeability than increasing fibre diameter. Permeability generally increased with aspect ratio and fibre content, whereas simply increasing dosage at relatively low aspect ratio was insufficient to produce a comparable permeability increase. Zhang and Tan [60] further compared PP fibres of different dimensions and found that 12 and 18 mm fibres and finer fibres of 28 and 33 μm were more effective in improving high-temperature permeability and spalling resistance than shorter or coarser fibres. Regardless of fibre dimensions, permeability increased markedly near approximately 150 °C. For a PP-fibre dosage of 3 kg/m^3^, that study recommended an aspect ratio of approximately 300 as an effective design scale for spalling mitigation.

Eidan et al. [36] confirmed the importance of fibre dimensions from the perspective of residual mechanics and cracking. In tests using 1–2 kg/m^3^ PP fibres with different lengths and exposure temperatures of 100–600 °C, 12 mm fibres generally outperformed 6 mm fibres. Approximately 400 °C was identified as a transition temperature beyond which the influence of PP fibres on residual strength changed markedly. A dosage of 0.1 vol.% already reduced cracking after heating and cooling to 600 °C, whereas the 12 mm, 0.2 vol.% system produced the best crack-control response. Nevertheless, the fibres could not prevent the substantial reduction in elastic modulus after exposure to 600 °C, demonstrating again that improvements in cracking and spalling resistance should not be interpreted as universal improvements in all residual mechanical properties.

Hybrid design should likewise be based on functional network formation rather than total fibre content. Heo et al. [61] compared combinations of PP, polyvinyl alcohol (PVA), cellulose, and nylon fibres under ISO 834 exposure. A combination of 9 mm nylon fibres and 19 mm PP fibres achieved spalling resistance comparable to that of a single-fibre system containing 0.10% PP, while using only approximately one-third of the total fibre content. The authors therefore proposed a fibre-effectiveness parameter based on the number of fibres per unit volume and fibre length. Together with subsequent PP–steel fibre investigations [8,46], these findings indicate that the number and connectivity of effective transport pathways are more physically meaningful variables for spalling control than total fibre mass alone.

### 6.3. Matrix Compactness and Moisture Condition Determine the Relative Importance of Fibres

The high-temperature function of fibres must be interpreted in the context of the surrounding matrix. PP fibres are particularly important in HSC and UHPC not because these materials require greater fibre-based load-carrying capacity, but because their low W/B, high powder content, and frequent use of silica fume produce dense microstructures with very low initial permeability. When heated moisture cannot escape rapidly, even a relatively small amount of PP fibres capable of forming a connected transport network can substantially alter pore-pressure development. In normal-strength concrete with higher initial transport capacity, the same PP network may provide a smaller marginal benefit [3,6,37,52,62].

Behnood and Ghandehari [37] investigated HSC with water-to-cementitious-material ratios of 0.40, 0.35, and 0.30, silica-fume replacement levels of 0, 6, and 10%, and PP-fibre dosages between 0 and 3 kg/m^3^, with exposure up to 600 °C. Severe strength loss occurred in all concretes after 600 °C, while denser, higher-strength silica-fume mixtures were more temperature-sensitive between 300 and 600 °C. Relative compressive strength was generally higher in PP-fibre concretes than in corresponding fibre-free mixtures, and 2 kg/m^3^ PP fibres provided a substantial benefit within the investigated system. The relevant conclusion is not that 2 kg/m^3^ represents a universal optimum, but that the marginal benefit of PP fibres became more pronounced as matrix compactness increased.

The W/B threshold observed by Choe et al. [52] further illustrates this coupling. Under rapid heating, HSC with W/B < 0.33 exhibited pronounced surface spalling and moisture-clogging characteristics, whereas slower heating changed the patterns of moisture redistribution and pore-pressure development. Matrix compactness alone therefore cannot predict spalling; it must be interpreted together with the timescale of thermal exposure.

Initial moisture condition determines the available vapour source. At a given matrix composition, higher saturation provides a greater quantity of evaporable water. If this coincides with low permeability and rapid heating, the risk of pore-pressure accumulation increases considerably. Yermak et al. [11] systematically varied aggregate type, moisture condition, and steel–PP fibre reinforcement in approximately 70 MPa HSC. Plain HSC did not spall under the tested conditions, whereas concrete containing 60 kg/m^3^ steel fibres did; the addition of PP fibres subsequently controlled macroscopic damage. These observations again demonstrate that crack restraint by steel fibres may reduce large-scale pressure-release pathways and that fewer visible cracks should not automatically be interpreted as lower spalling risk in dense, moisture-containing matrices.

Coarse aggregates can also contribute to the transport network in UHPC through thermally induced mismatch cracking. Li et al. [46,59] showed that increasing aggregate size alone produced only an approximately twofold permeability increase, whereas combining larger aggregates with PP fibres increased permeability by roughly three orders of magnitude. Aggregate size should therefore not be treated as an independent “enhancing factor”; its contribution depends on whether aggregate-induced cracking connects with PP-fibre-generated channels to form a continuous transport network. The matrix, aggregate, and fibre system should consequently be regarded as an integrated percolation topology rather than as three mix-design parameters whose effects can be linearly superimposed.

Glass-fibre and steel–glass hybrid systems likewise exhibit strong matrix dependence. Kumari et al. [7] compared steel, glass, and steel–glass hybrid fibres in M40 and M80 self-compacting concretes subjected either to 1, 3, 14, and 28 thermal cycles at 200 °C or to single exposures at 200, 400, and 600 °C. The hybrid steel–glass systems generally showed better resistance to thermal damage than their single-fibre counterparts, but the magnitude of this advantage depended on both matrix strength grade and thermal history. Accordingly, conclusions regarding the high-temperature function of glass fibres should not be directly extrapolated from RPC or a single SCC system to all concrete types.

### 6.4. Multi-Factor Coupling and the Evidence Boundary of Cross-Study Comparisons

The preceding evidence demonstrates that no fixed “best fibre system” exists independently of experimental conditions. Han et al. [54] found that 0.05 vol.% PP fibres were sufficient to eliminate spalling in their HPC system, whereas Behnood and Ghandehari [37] identified 2 kg/m^3^ as beneficial for residual performance in another HSC system. UHPC studies further indicate that the effectiveness of 3 kg/m^3^ PP fibres depends strongly on fibre dimensions and transport-network connectivity [9,46,60]. These values are not contradictory because they correspond to different matrices, performance objectives, and thermal exposure paths. The purpose of a review should therefore not be to average such values into a nominal universal optimum, but to identify the controlling variables underlying them.

The same caution applies to steel fibres. They should not be classified simply as a uniformly positive variable for fire resistance. Steel fibres can improve post-cracking load transfer, tensile resistance, and toughness, yet Algourdin et al. [45] and Yermak et al. [11] both reported increased spalling susceptibility in high-steel-fibre systems under certain conditions, whereas PP-fibre addition restored effective spalling control. This behaviour is consistent with the functional distinction established in Section 3 and Section 5: steel fibres primarily control macrocracking and post-cracking load transfer, whereas PP fibres primarily enhance internal transport. In dense concrete, the optimum combination therefore depends on balancing structural integrity against vapour-pressure release.

Scale and boundary conditions impose additional limitations on material-level data. Li and Zhang [48] showed that lateral restraint in PP–steel-fibre UHPC simultaneously modifies thermal stress, pore pressure, and spalling development. Khaliq and Kodur [28], through direct high-temperature measurements of thermal and mechanical properties from 20 to 800 °C, further demonstrated that hot-state measurements and residual tests after cooling represent physically different material states. Colombo et al. [63] reached a similar conclusion in comparisons of hot-state and residual SFRC behaviour. A reported “property at 600 °C” must therefore specify whether the material was tested at 600 °C or after cooling from 600 °C.

For cross-study comparisons, two principles are consequently recommended. First, normalised residual properties, spalling occurrence, spalling depth, peak pore pressure, and permeability changes should be prioritised over direct pooling of absolute mechanical properties from concretes with different strength classes. Second, every quantitative conclusion should be accompanied by a minimum set of experimental metadata, including matrix W/B and initial strength, actual moisture condition, fibre material and geometry, individual dosage of each fibre type, heating curve, peak temperature, holding duration, cooling regime, specimen dimensions, and whether the test was performed in the hot state or after cooling. Data lacking this information may still support qualitative trend identification but should not be used to derive universal optimum dosages or quantitative empirical models.

Representative quantitative evidence supporting these conclusions is summarised in Table 5, while Table 6 provides evidence-based rules for comparing high-temperature FRC studies across different material and testing conditions.

As shown in Table 5 and Table 6, the most robust conclusions are not associated with a fixed “optimum fibre dosage” but with several repeatedly supported mechanistic relationships. Dense matrices and rapid heating increase the risk of restricted vapour transport; PP-fibre effectiveness depends on the formation of a sufficiently connected transport network; the principal contribution of steel fibres lies in post-cracking load transfer rather than direct pressure relief; the advantages of hybrid systems arise from functional complementarity rather than from increasing the total fibre content; and cooling and testing state can substantially alter the measured “residual” response. These relationships provide a more defensible basis for engineering interpretation than isolated optimum values reported in individual experiments.

Accordingly, future research on high-temperature FRC should move from single-variable dosage optimisation towards the integrated design of thermal path, fibre-network architecture, and matrix state. For spalling mitigation, priority should be given to characterising the number density, geometric scale, three-dimensional connectivity, and temperature-dependent permeability associated with PP or other low-melting-point fibres. For post-fire residual load-bearing performance, the fibre–matrix bond, residual tensile and flexural response, and toughness of load-bearing fibres such as steel fibres should be evaluated simultaneously. Only by integrating these transport and mechanical functions can high-temperature fibre design progress from empirical dosage selection towards a physically grounded and transferable framework.

## 7. Research Challenges and Future Directions

Existing research has established a relatively systematic understanding of how steel, polypropylene (PP), glass, and hybrid fibres influence thermal damage, residual mechanical properties, and spalling behaviour in concrete exposed to elevated temperatures. Nevertheless, most available conclusions are still derived from independent experiments conducted using different material systems, specimen scales, and thermal exposure regimes, resulting in strong condition dependence. In particular, questions such as the “optimum fibre dosage”, whether a given fibre type can reliably suppress spalling, and which fibre system provides the best overall high-temperature performance remain difficult to answer in a manner that can be directly generalised across different concrete matrices and structural applications [45,46,48,52].

Al-Bashiti and Naser [64] compiled 1069 concrete fire-spalling tests and evaluated 43 material, geometric, mechanical, and environmental variables. Their analysis showed that the existing experimental database is strongly concentrated within a limited number of material systems and parameter ranges, while substantial inconsistencies remain in testing conditions and reporting practices among different studies. This finding suggests that future progress should not rely primarily on increasing the number of combinations of fibre type, dosage, and temperature. Instead, greater emphasis should be placed on improving experimental comparability, obtaining more direct mechanistic evidence, and strengthening the reliability of translating material-level findings to structural-scale applications.

**Figure 5:**
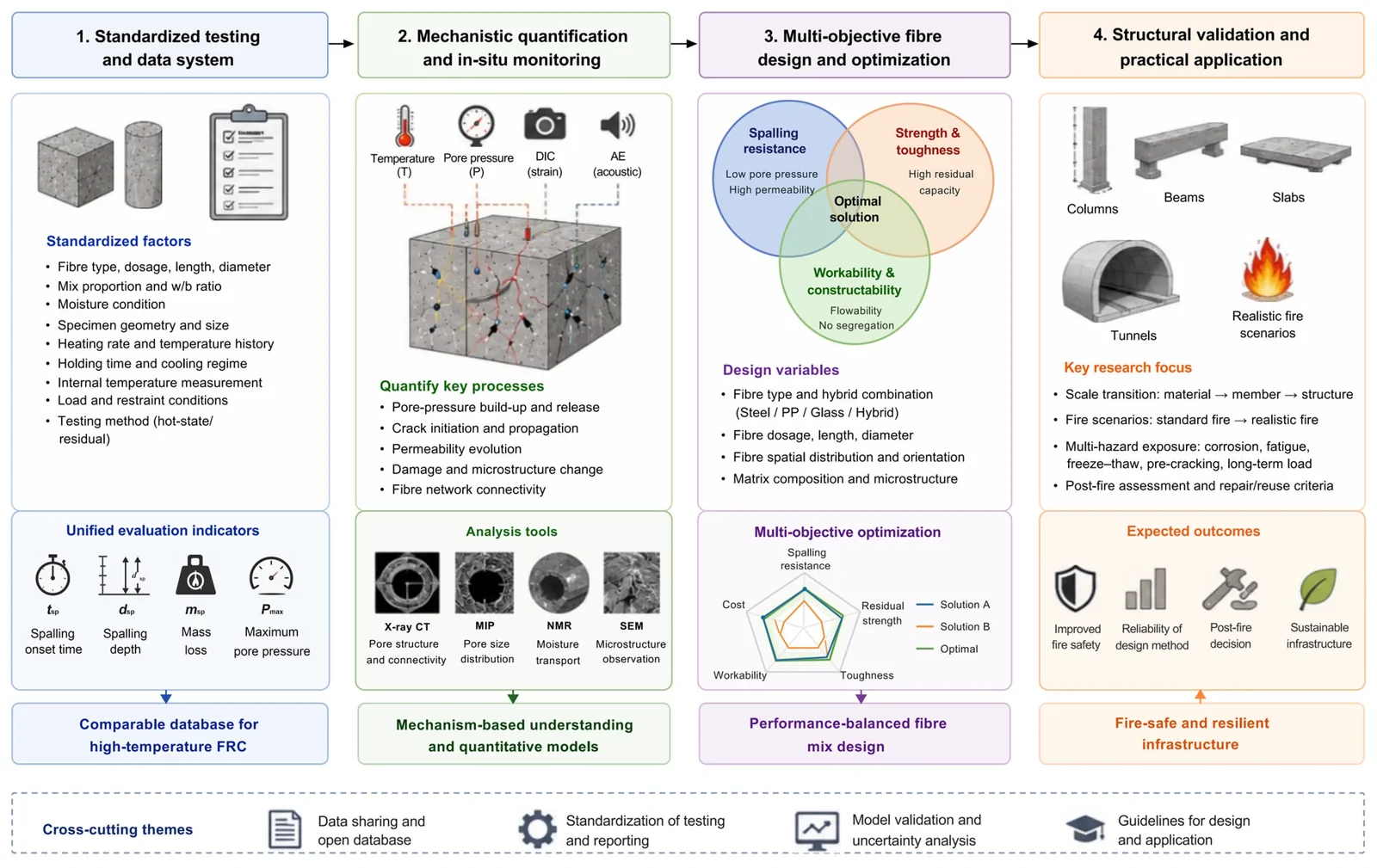
Future research framework for fibre-reinforced concrete subjected to elevated temperatures.

Based on the evidence reviewed in the preceding sections, future research can be organised into four closely related directions: establishing comparable testing and data frameworks; moving from before-and-after performance comparison towards time-resolved quantification of high-temperature mechanisms; shifting from single-property optimisation towards multi-objective hybrid-fibre design; and extending investigations from small-scale material specimens to realistic structures and complex service environments. These research priorities and their interrelationships are schematically summarised in Figure 5.

### 7.1. Establishing Comparable High-Temperature Testing and Data Frameworks

One of the most immediate limitations of the current literature is the lack of consistency in thermal exposure conditions and experimental metadata. Studies described simply as “600 °C tests” may differ substantially in heating rate, holding duration, actual internal specimen temperature, initial moisture condition, and cooling regime. Algourdin et al. [45] showed that the spalling response of the same steel–PP fibre system changed when ISO 834 fire exposure was compared with different constant heating rates. Choe et al. [52], using concretes with W/B values of 0.55, 0.33, 0.18, and 0.125, further demonstrated that W/B and heating rate jointly influence moisture migration, pore-pressure development, and spalling mode. Peak temperature alone is therefore insufficient to define a high-temperature exposure condition.

Future studies should first establish a standardised set of minimum reporting requirements, rather than requiring all researchers to adopt identical test protocols. At a minimum, studies should report fibre type, volume fraction, length, and diameter; matrix W/B and strength; pre-test moisture condition; specimen dimensions and heated surface; heating curve and heating rate; peak temperature and holding duration; cooling regime; and whether the mechanical test was performed in the hot state or after cooling. For structural-member tests, applied load level and restraint condition should also be reported. Only when these metadata are available can cross-study comparisons be placed on a physically meaningful basis. Accordingly, future reporting frameworks should build on the existing RILEM TC 256-SPF recommendations [50,51], while retaining fibre-specific descriptors and coupled mechanical–transport variables that are particularly relevant to FRC.

Spalling-related metrics likewise require greater standardisation. A considerable proportion of the literature still relies primarily on a binary “spalling/no-spalling” outcome, which cannot distinguish minor surface detachment from severe spalling associated with substantial loss of effective cross-section. Future studies should, wherever possible, record spalling onset time, maximum spalling depth, spalled mass or area, peak pore pressure, and residual cross-sectional dimensions. Such measurements would allow the assessment to move from merely determining whether spalling occurred to quantifying its severity and structural significance.

Hot-state properties and residual properties after cooling should also be treated as separate data categories. Colombo et al. [63] directly compared the hot-state and residual tensile responses of steel-fibre-reinforced concrete and demonstrated that the two test conditions incorporate different thermal-stress and cooling-damage histories and therefore should not be pooled without distinction. Future databases and empirical models should consequently store at least four independent categories of information: material composition, thermal history, fibre architecture, and measured response.

### 7.2. From End-State Comparison to Time-Resolved Quantification of High-Temperature Mechanisms

Current research has established the broad understanding that PP fibres facilitate pore-pressure relief, whereas steel fibres contribute primarily to crack bridging. However, much of the supporting evidence is still derived from comparisons between pre-heating and post-heating states. A common experimental procedure involves heating, cooling, residual-strength testing, and subsequent SEM or MIP characterisation. Such approaches are useful for identifying the final damage state but are less effective in determining when pore connectivity, crack initiation, and pore-pressure accumulation occur, or which process actually triggers macroscopic spalling.

Li and Zhang [48] simultaneously examined pore pressure, thermal stress, and lateral restraint in UHPC and showed that spalling cannot be adequately explained by a single criterion based on pore pressure exceeding tensile strength. Thermal stress, material degradation, and pore-pressure development must instead be considered together. Toropovs et al. [22], using neutron imaging to obtain simultaneous information on internal temperature, pressure, and moisture distribution in heated HPC, further demonstrated the pronounced spatial and temporal evolution of moisture redistribution and pore-pressure development. These findings indicate that future experiments should prioritise synchronous measurements of temperature fields, pore-pressure fields, crack evolution, and permeability rather than relying only on final residual properties.

PP-fibre research, in particular, should move from a dosage-based description towards a spatial-network description. Li et al. [9] systematically investigated the effects of fibre geometry and dosage on the post-heating permeability of UHPC, while Zhang et al. [10] further quantified the three-dimensional distribution of polymer fibres using X-ray computed tomography. In the latter experimental system, aspect ratios of approximately 300–600 and fibre volume fractions of approximately 0.3–0.4% were identified as an effective range for spalling mitigation. The significance of these results does not lie in defining a universally applicable dosage window but in demonstrating that fibre number density, three-dimensional distribution, and network connectivity are more physically meaningful descriptors of transport performance than volume fraction alone.

Synthetic fibres should likewise not be classified solely according to melting temperature. Lin et al. [65] compared multiple synthetic fibres under similar high-temperature conditions and showed that thermal-expansion behaviour, melting characteristics, and the resulting differences in internal pore structure all influence spalling resistance in UHPC. Future studies should therefore combine X-ray CT, mercury intrusion porosimetry (MIP) or nuclear magnetic resonance (NMR), digital image correlation (DIC), acoustic emission (AE), and synchronised temperature–pore-pressure measurements to establish a continuous evidence chain linking fibre thermal response, pore-network evolution, crack development, and eventual spalling.

Future studies on steel fibres should similarly place greater emphasis on the high-temperature fibre–matrix interface and pull-out mechanism. Ruano et al. [26] demonstrated that elevated temperatures modify the pull-out behaviour of steel fibres from high-strength concrete and that this degradation directly affects residual post-cracking performance. Accordingly, future steel-fibre studies should not be limited to reporting residual compressive or flexural strength but should link single-fibre bond behaviour, fibre orientation, and bridging efficiency to macroscopic toughness and fracture response.

### 7.3. From a Single Optimum Dosage to Multi-Objective Hybrid-Fibre Design

A large proportion of existing high-temperature FRC studies still aim to identify an “optimum fibre dosage”. However, the optimum value depends fundamentally on the performance objective being considered. Steel fibres can improve post-cracking load transfer and toughness but do not necessarily reduce pore pressure, whereas PP fibres can mitigate spalling but may introduce additional porosity after melting, potentially reducing residual stiffness and strength. High-temperature fibre design is therefore inherently a multi-objective optimisation problem involving trade-offs among spalling resistance, residual strength, toughness, workability, and material cost.

Shen et al. [66], in an optimisation study of steel–PP hybrid-fibre concrete, found that 96% of pore-pressure peaks occurred within approximately 150–250 °C and explicitly treated the mechanical contribution of steel fibres and the pore-pressure-control function of fine PP fibres as separate design objectives. This study illustrates that the rationale of hybridisation lies in functional differentiation rather than simply increasing the total fibre volume fraction.

Evidence of a genuine mesoscopic interaction between steel and PP fibres has been reported. Li et al. [8] showed that channels produced after PP-fibre melting can become more strongly interconnected through thermally induced microcracks around both steel and PP fibres, thereby increasing high-temperature permeability and reducing spalling susceptibility. At the same time, steel fibres remain capable of bridging cracks and transferring stress after cracking. Future research on hybrid fibres should therefore directly verify whether improvements in transport-network connectivity and retention of post-cracking load transfer occur simultaneously. The term synergistic effect should not be used merely because a hybrid mixture produces a higher final performance value than a corresponding single-fibre mixture.

Current hybrid-fibre research is still strongly concentrated on steel–PP systems, whereas the high-temperature evidence base for glass fibres remains relatively limited. Wang et al. [38] and Kumari et al. [7] indicate that glass fibres and steel–glass hybrid systems can contribute to crack control and residual-property retention within certain temperature ranges, but fibre–matrix bond degradation, cumulative damage under thermal cycling, and multiscale crack-bridging mechanisms remain insufficiently quantified. Future work could therefore compare steel–PP, steel–glass, and ternary hybrid systems, provided that the design objective is clearly specified in advance—whether the aim is spalling mitigation, toughness enhancement, residual-strength retention, or multi-performance optimisation.

Natural and bio-based fibres also represent an emerging reinforcement option for more sustainable cementitious composites. Their renewable origin and potential contribution to lower-carbon cement-based materials have attracted increasing attention, although their incorporation may also alter fresh-state and rheological behaviour [67]. However, their performance under elevated-temperature conditions remains insufficiently understood. In particular, thermal degradation of the fibres, moisture sensitivity, changes in fibre–matrix interfacial bonding, and the resulting effects on residual mechanical performance and durability require further systematic investigation. Future studies should therefore evaluate not only their mechanical contribution under ambient conditions but also their thermal stability, post-heating residual performance, and long-term durability after elevated-temperature exposure.

### 7.4. Extending Research from Material Specimens to Structural Scale and Realistic Service Conditions

Most current studies are still based on cubes, cylinders, or small beam specimens, whereas engineering applications ultimately concern structural members such as columns, beams, slabs, walls, and tunnel linings. Material-scale tests are appropriate for isolating fibre-related mechanisms but cannot directly determine structural fire-resistance duration. Structural members are additionally subjected to thermal gradients, reinforcement restraint, axial loading, eccentricity, and boundary constraints, all of which may significantly alter cracking and spalling behaviour.

Li, Du, and Tan [68] conducted constant-load fire tests on five UHPC columns and one HSC column to investigate the effects of concrete strength, fibre configuration, and load eccentricity. The results showed that UHPC columns containing hybrid steel–PP fibres experienced only limited spalling under concentric compression, whereas eccentric loading intensified spalling on the compression side and reduced overall fire performance. Khaliq and Kodur [32] similarly compared HSC columns containing no fibres, PP fibres, steel fibres, and hybrid fibres and found that the hybrid-fibre columns exhibited better overall fire performance than the single-fibre and fibre-free columns under their test conditions. These studies demonstrate that material-level fibre benefits must be validated at the member scale before they can be translated into reliable structural design recommendations.

A second necessary extension concerns realistic fire exposure scenarios. Many existing studies employ constant furnace temperatures or the ISO 834 standard fire curve, whereas tunnel fires, petrochemical fires, and transportation-related fires may involve much more rapid heating rates, localised heating, and different exposure durations. Beyond standardised benchmark tests, future work should therefore include rapid-heating and localised-fire scenarios tailored to specific engineering applications, particularly to determine whether PP-fibre-related transport pathways can develop before critical pore pressure is reached.

A third direction is the transition from single-fire exposure to multi-hazard and pre-damaged service conditions. Real structures may already have experienced corrosion, fatigue, freeze–thaw cycling, pre-cracking, or long-term mechanical loading before a fire occurs. Their initial crack patterns, reinforcement bond conditions, and permeability states therefore differ substantially from those of pristine laboratory specimens. Chandra and Sharma [69], in a 2025 study on corroded reinforced-concrete columns containing hybrid steel–PP fibres, reported that fibre reinforcement improved the performance of corrosion-damaged columns and delayed or mitigated fire-induced spalling. This type of investigation represents an important transition from the idealised scenario of an initially undamaged structure exposed to a single fire towards the more realistic case of pre-damaged infrastructure subsequently subjected to fire.

Future high-temperature FRC research should therefore progressively establish a continuous link from material composition and fire exposure to residual material properties, structural residual capacity, and post-fire repair decisions. From an engineering perspective, the ultimate question is not simply how much strength a fibre-reinforced concrete retains after exposure to 600 °C, but whether a structure subjected to a specific fire and prior service damage remains safe for continued use, what level of repair is required, and how much residual safety margin remains.

The principal research gaps identified above and the corresponding recommended directions are summarised in Table 7.

Overall, the central challenge for future research on fibre-reinforced concrete at elevated temperatures is not to continue expanding the number of combinations of fibre type × dosage × temperature, but to improve comparability, mechanistic observability, and structural applicability. Standardised testing and reporting frameworks are required to enable meaningful comparisons among studies; in situ and three-dimensional characterisation methods are needed to transform qualitative mechanisms into quantifiable ones; multi-objective hybrid-fibre design is necessary to avoid optimisation of a single response at the expense of others; and structural-scale and multi-hazard investigations are essential for determining whether material-level advances can be translated into reliable fire design and post-fire assessment.

## 8. Conclusions

This review examined the high-temperature behaviour of fibre-reinforced concrete from the perspectives of damage mechanisms, fibre-specific functions, residual mechanical properties, cracking and spalling, governing factors, and future research needs. The available evidence shows that the thermal response of FRC is controlled by the coupled effects of matrix degradation, moisture and vapour transport, crack evolution, fibre–matrix interaction, and thermal exposure history.

Different fibres perform distinct functions at elevated temperatures. Steel fibres mainly contribute through crack bridging, stress redistribution, and post-cracking load transfer, and are therefore particularly effective in improving residual tensile, flexural, and toughness-related performance. PP fibres primarily enhance permeability and vapour transport through interfacial damage, softening, and melting, thereby reducing pore-pressure accumulation and spalling risk. Glass fibres can provide crack-control effects within a certain temperature range, although the available evidence remains relatively limited. Hybrid systems, especially steel–PP combinations, can provide complementary functions by combining pressure relief with post-cracking load transfer, although their effectiveness remains dependent on fibre configuration, matrix characteristics, and thermal exposure conditions.

Residual compressive strength alone is insufficient to evaluate high-temperature FRC. A more representative assessment should also consider tensile and flexural behaviour, elastic modulus, toughness, fracture energy, and spalling severity. Fibre effectiveness is strongly influenced by heating rate, cooling regime, moisture condition, matrix compactness, fibre geometry, spatial distribution, and specimen scale; therefore, a universal optimum fibre dosage is not supported by current evidence.

Overall, fibres do not eliminate thermal damage but modify its development and the residual load-bearing mechanism. Future research should focus on standardised test reporting, time-resolved characterisation of crack–pore-pressure–permeability evolution, multi-objective hybrid-fibre design, and structural-scale validation under realistic fire and multi-hazard conditions.

## Figures and Tables

**Table 1:** Representative experimental studies on the high-temperature mechanisms of fibre-reinforced concrete.

Ref.	Fibre System	Concrete Matrix	Thermal Condition	Main Focus	Main Mechanistic Implication
[25]	Steel	NSC, MSC, HSC, UHPM	Elevated-temperature pull-out tests	Straight and hooked-end fibre bond	Fibre geometry and matrix strength control high-temperature pull-out behaviour
[26]	Steel	HSC	Up to 500 °C	Fibre pull-out and residual mechanical behaviour	Degradation of fibre–matrix bond contributes directly to the loss of post-cracking capacity
[27]	3D/4D/5D steel	Concrete	300, 500 and 800 °C	Residual strength and toughness	Steel-fibre geometry remains important after thermal exposure
[3]	PP	HPC	High-temperature exposure	Post-heating permeability/microstructure	PP fibres reduce pore-pressure development and increase permeability
[6]	PP	In situ concrete	Fire/high-temperature exposure	Permeability and spalling	Increased permeability is a central mechanism of PP-fibre-induced spalling mitigation
[29]	PP	HSC	20–300 °C	In situ high-temperature permeability	Permeability may increase markedly before complete PP-fibre melting
[9]	PP	UHPC	Heat exposure; post-heating permeability	Fibre geometry, dosage and permeability	Fibre dimensions and content govern transport-network formation
[10]	Polymer/PP	UHPC	High-temperature exposure	3D fibre distribution and spalling	Aspect ratio, number density and spatial connectivity govern percolation behaviour
[30]	Glass	RPC	200–800 °C	Residual mechanical properties and spalling	Glass-fibre effectiveness is strongly dependent on temperature and dosage
[7]	Steel + glass	SCC	Thermal cycles and 200–600 °C exposure	Residual strength	Hybrid performance depends on thermal history as well as fibre combination
[8]	Steel + PP	UHPC	Elevated-temperature exposure	Spalling, permeability and microstructure	Steel-induced cracking can improve connectivity between PP-fibre-generated channels
[32]	Steel, PP, hybrid	HSC columns	Fire exposure	Structural fire resistance	Material-scale fibre mechanisms interact with structural restraint and loading

**Table 2:** Representative experimental studies on the residual mechanical properties of fibre-reinforced concrete after elevated-temperature exposure.

Ref.	Fibre System	Matrix	Temperature	Testing State/Cooling Regime	Main Properties Investigated	Main Significance
[33]	Steel	HPC	105–1200 °C	Post-cooling residual testing; cooling regime not clearly reported in accessible source	Strength and stiffness	Establishes broad temperature-dependent behaviour of steel-FRC
[34]	Steel	RPC	20–800 °C	Post-cooling residual testing; cooling regime not clearly reported in accessible source	Compressive and tensile properties	Demonstrates effects of fibre content, temperature, holding time, and specimen size
[28]	Steel/PP systems	HPSCC	20–800 °C	Hot-state testing at target temperature; no cooling involved	Compressive strength, tensile strength, elastic modulus	Provides simultaneous thermal and mechanical property measurements
[27]	3D/4D/5D steel	Concrete	20, 300, 500, 800 °C	Post-cooling residual testing; cooling regime not clearly reported in accessible source	Compressive strength, flexural strength, toughness	Demonstrates importance of fibre geometry and anchorage
[36]	PP	Concrete	Multiple temperatures	Post-cooling residual testing; specimens cooled to ambient temperature; specific cooling method not clearly reported in accessible source	Strength and elastic modulus	Shows dependence on fibre length and dosage and severe modulus degradation
[37]	PP	HSC	Up to 600 °C	Post-cooling residual testing; cooling regime not clearly reported in accessible source	Compressive and splitting tensile strength	Demonstrates different temperature sensitivity of compression and tension
[38]	Glass	Concrete	440–1000 °C	Post-exposure residual testing; cooling regime not clearly reported in accessible source	Compressive strength and thermal conductivity	Shows effects of glass-fibre length and dosage
[23]	Steel + PP	HSC	20–800 °C	Post-cooling residual testing; cooling regime not clearly reported in accessible source	Compressive and splitting tensile strength	Demonstrates complementary roles of low- and high-melting-point fibres
[39]	Steel	Normal- and lightweight concrete	200–800 °C	Hot-state testing at target temperature; no cooling involved	Residual flexural tensile strength	Directly evaluates post-cracking residual flexural capacity
[40]	PP	HSC	Elevated temperatures	Post-cooling residual testing; cooling regime not clearly reported in accessible source	Compressive and fracture properties	Demonstrates interaction between PP-fibre length, content, and fracture behaviour
[42]	Steel	Concrete	200–1000 °C	Post-cooling residual testing; cooling regime not clearly reported in accessible source	Compressive, flexural, and toughness properties	Extends evaluation from peak strength to energy-based toughness
[43]	Steel + PP	SCHPC	Elevated temperatures	Post-cooling residual testing; cooling regime not clearly reported in accessible source	Compressive, flexural, fracture energy, and toughness	Demonstrates the value of fibre-cocktail design

**Table 3:** Qualitative mechanism-based comparison of fibre contributions to residual mechanical performance.

Property	Steel Fibre	PP Fibre	Glass Fibre	Hybrid Fibres
Compressive strength	Mainly indirect improvement through crack restraint and integrity retention	Variable; improved integrity may coexist with additional post-melting porosity	Temperature- and dosage-dependent	Can combine spalling mitigation with crack restraint
Tensile strength	Direct contribution through fibre bridging while the fibre–matrix bond remains effective	Limited after fibre melting	Crack-bridging contribution at suitable temperatures	High potential when a load-bearing fibre is retained
Flexural strength	Strong crack-bridging and residual load-transfer effect	Limited direct contribution after melting	Moderate crack-control potential	Favourable when steel or another stable fibre provides bridging
Elastic modulus	Limited ability to prevent matrix-dominated stiffness degradation	Limited	Limited evidence	Matrix degradation remains dominant
Toughness/fracture energy	Important contribution through pull-out and bridging	Strongly temperature-dependent	Potential contribution, but evidence remains limited	Potential benefit through complementary mechanisms
Preservation against catastrophic spalling damage	Mainly indirect	Primary contribution	Not the primary mechanism	Particularly relevant for steel–PP systems

Note: The descriptors in this table represent qualitative interpretations based on the overall trends reported in the reviewed literature and should not be interpreted as quantitative rankings or effect-size estimates. Representative quantitative evidence for selected fibre systems and thermal conditions is provided in Table 5.

**Table 4:** Representative studies on cracking and spalling of fibre-reinforced concrete at elevated temperatures.

Ref.	Fibre System	Concrete	Main Variables	Main Observations Relevant to Cracking/Spalling
[3]	PP	HPC	PP dosage, temperature	PP fibres markedly altered permeability near their melting range and reduced spalling susceptibility
[6]	PP	In situ concrete	PP content, fire exposure	Increasing PP content reduced spalling through permeability enhancement observed under the reported high-temperature test conditions
[44]	PP and other organic fibres	HPC	Fibre type	Fibre thermal behaviour and interface changes strongly affected pore pressure and spalling
[24]	Steel + PP	HSC	Fibre cocktail	Combined fibres improved overall high-temperature behaviour through complementary functions
[11]	Steel, PP, steel + PP	HSC	Fibre type, moisture, aggregate	Steel fibres controlled crack opening; PP fibres were more effective in preventing macroscopic spalling
[45]	Steel, PP, steel + PP	Concrete	Heating rate	Spalling response depended strongly on heating regime; PP addition prevented spalling in the tested hybrid mixtures
[30]	Glass	RPC	2–4% vol. GF, 200–800 °C	Glass fibres reduced spalling in the investigated RPC, but evidence remains matrix-specific
[46]	Steel + PP	UHPC	Aggregate size and fibres	Fibre- and aggregate-induced crack networks influenced pore pressure and explosive spalling
[47]	Hybrid fibres	HSC	Heating rate, measurement depth	Hybrid reinforcement modified internal pore-pressure development
[8]	Steel + PP	UHPC	Fibre combination	PP-generated tunnels and thermally induced microcracks formed a more connected permeability network
[48]	Steel + PP	UHPC	Lateral restraint	Mechanical restraint altered thermal stress, pore pressure, and spalling response
[32]	Steel, PP, hybrid	HSC columns	Structural fire exposure	Hybrid-fibre HSC columns showed the best overall fire response among the tested fibre systems
[49]	PP–steel fibre	SCC	Fibre type, elevated temperature	PP fibres reduced explosive spalling, whereas residual mechanical effects differed between fibre systems

**Table 5:** Quantitative evidence for key parameters governing the high-temperature response of fibre-reinforced concrete.

Study	Material/Variable	Thermal Condition	Quantitative/Direct Observation	Interpretation
Algourdin et al. [45]	HSC; plain/60 kg/m^3^ SF/60 kg/m^3^ SF + 0.75 kg/m^3^ PP	10 °C/min and ISO 834	60 kg/m^3^ SF aggravated spalling, especially under ISO 834; adding 0.75 kg/m^3^ PP prevented spalling under both thermal loadings	Heating regime changes the apparent benefit of the same fibre system
Choe et al. [52]	HSC with different W/B	Fast vs. slow heating	Under fast heating, HSC with W/B < 0.33 exhibited surface spalling associated with moisture clogging	Matrix compactness and heating rate must be considered together
Peng et al. [53]	Hybrid-fibre HPC; 200–800 °C	Natural cooling; water spray 5–60 min; quenching	Water cooling caused larger losses in compressive/tensile strength and fracture energy; ≥30 min spraying approached quenching damage	Cooling is part of the thermal history, not a post-test detail
Han et al. [54]	HPC; W/B 0.30/0.40; PP 0–0.10 vol.%	Fire-resistance test	All no-PP specimens spalled; ≥0.05 vol.% PP prevented spalling. PP-only residual compressive strength ≈ 70%; PP + metal fabric ≈ 90%	A small effective PP network can preserve integrity; strength retention is a separate metric
Li et al. [46]	UHPC; PP, SF, and aggregate-size combinations	One-sided heating at 2 °C/min; post-heating residual permeability	3 kg/m^3^ PP raised intrinsic residual permeability from 0.733 to 13.8 × 10^−18^ m^2^ (~19×); PP + large aggregate ≈ 1500×; PP + SF ≈ 1040×	Connectivity between fibre tunnels and thermally induced cracks dominates transport
Li et al. [9]	15 PP-UHPC mixtures	Geometry and dosage varied	Increasing fibre length and dosage affected permeability more strongly than diameter; at low aspect ratio, dosage increase alone was ineffective	Fibre content cannot replace geometry/network descriptors
Zhang and Tan [60]	UHPC; 3 kg/m^3^ PP	Various PP dimensions; ISO 834	12/18 mm long and 28/33 μm fine fibres were more effective; permeability increased markedly near 150 °C; aspect ratio ≈ 300 recommended	Critical dimensions govern early formation of pressure-release paths
Eidan et al. [36]	PP-FRC; 1–2 kg/m^3^; 6/12 mm	100–600 °C	12 mm fibres outperformed 6 mm fibres; ~400 °C was a critical transition; 0.1 vol.% reduced cracking to 600 °C and 12 mm–0.2 vol.% was best for crack prevention	Dosage, length, and target response must be optimised jointly
Behnood and Ghandehari [37]	HSC; W/CM 0.30–0.40; silica fume 0–10%; PP 0–3 kg/m^3^	Up to 600 °C	300–600 °C was critical for higher-strength mixtures; 2 kg/m^3^ PP improved residual properties in the tested HSC	A useful PP dosage is matrix-specific rather than universal
Heo et al. [61]	Concrete with PP, nylon, PVA, and cellulose fibres	ISO 834	9 mm nylon + 19 mm PP achieved comparable spalling protection using about one-third of the fibre content required by single 0.10% PP	Hybridisation can reduce total fibre demand by increasing fibre effectiveness
Yermak et al. [11]	~70 MPa HSC; steel and/or PP	ISO 834	Plain concrete did not spall, whereas 60 kg/m^3^ SF concrete did; PP addition controlled macroscopic spalling	Crack restraint by SF and pressure relief by PP are different functions
Kumari et al. [7]	M40/M80 SCC; steel, glass, and hybrid steel–glass	200 °C × 1/3/14/28 cycles; 200/400/600 °C	Hybrid steel–glass SCC showed better overall thermal resistance than single-fibre counterparts, with response dependent on matrix grade and thermal history	Hybrid benefit is not transferable without matching matrix and exposure conditions

Note: The quantitative values listed in Table 5 correspond to the specific mixtures and testing protocols of the original studies and should not be interpreted as universal design thresholds applicable across different concrete systems.

**Table 6:** Evidence-based rules for comparing high-temperature FRC studies.

Comparison Variable	Recommended Rule	Evidence/Reason
Peak temperature	Compare only when heating rate/fire curve and holding duration are similar	The same nominal “600 °C” may represent substantially different thermal gradients and pore-pressure histories [45,52,53]
PP dosage	Do not compare kg/m^3^ alone; report volume fraction, length, diameter, and number density	0.05 vol.% was sufficient in Han et al. [54], whereas other HSC/UHPC systems required different contents [9,36,37,60]
Steel-fibre content	Separate post-cracking benefit from anti-spalling performance	60 kg/m^3^ SF improved mechanical integrity but increased spalling susceptibility in some dense concretes [11,45]
Hybrid fibres	Report each fibre component separately and identify its targeted function	Steel–PP systems couple post-cracking load transfer with pressure relief; other hybrid systems may operate through different mechanisms [8,24,46,61]
Matrix compactness	Always report W/B, strength class, and major supplementary cementitious-material content	Rapidly heated HSC with W/B < 0.33 showed moisture-clog-related spalling [52]; silica-fume HSC was highly temperature-sensitive [37]
Moisture condition	Pre-drying/conditioning should be reported for meaningful spalling comparisons	Available vapour source interacts with permeability and heating rate; moisture cannot be treated as a secondary specimen detail [47,52,62]
Cooling regime	Separate residual-property datasets according to cooling method	Water spraying for ≥30 min approached quenching-induced damage in Peng et al. [53]
Test state	Do not pool hot-state and post-cooling residual properties	They represent different material states and may produce different tensile and post-cracking responses [28,63]

**Table 7:** Key research gaps and recommended future directions for fibre-reinforced concrete at elevated temperatures.

Research Issue	Current Limitation	Recommended Future Work	Expected Outcome
Experimental comparability	Heating, moisture, cooling, and specimen conditions vary widely	Establish standardised minimum metadata and a common reporting framework	Reliable cross-study comparison
Spalling evaluation	Spalling is often reduced to a binary spalling/no-spalling classification	Report onset time, depth, mass/area loss, pore pressure, and remaining cross-section	Severity-based fire assessment
Mechanism identification	Evidence is largely based on before/after observations	Synchronise temperature, pore pressure, three-dimensional pore structure, and crack monitoring	Time-resolved thermo-hydro-mechanical evidence
PP-fibre design	Dosage is often treated as the primary design variable	Quantify fibre geometry, number density, orientation, and network connectivity	Connectivity-based anti-spalling design
Steel-fibre design	Emphasis remains on volume fraction and peak strength	Relate high-temperature bond, pull-out behaviour, and fibre orientation to post-cracking response	Improved prediction of residual toughness
Glass fibres	Evidence remains limited and strongly matrix-specific	Conduct systematic bond, thermal-cycling, and fracture studies	Clearer range of applicability
Hybrid fibres	Synergy is often inferred only from final properties	Directly quantify interactions between transport regulation and crack bridging	Mechanistically justified hybrid design
Mix optimisation	Single-property optimisation remains common	Apply multi-objective optimisation to spalling resistance, strength, toughness, and workability	Performance-balanced fibre mixtures
Scale transition	Most evidence is derived from small material specimens	Conduct full-scale tests on beams, columns, slabs, and tunnel linings	Structural validation
Fire scenario	Furnace heating and ISO fire exposure dominate	Include rapid, localised, and application-specific fire curves	More realistic design data
Multi-hazard exposure	Fresh, undamaged specimens dominate current testing	Investigate corrosion–fire, fatigue–fire, freeze–thaw–fire, and pre-cracked–fire scenarios	Infrastructure-relevant resilience assessment
Post-fire assessment	Residual strength is often treated as the final outcome	Link residual properties to structural re-use and repair criteria	Rational post-fire decision framework

## Data Availability

No new data were created or analyzed in this study. Data sharing is not applicable to this article.
